# Socially interdependent risk taking

**DOI:** 10.1007/s11238-023-09927-x

**Published:** 2023-04-19

**Authors:** Alexandros Karakostas, Giles Morgan, Daniel John Zizzo

**Affiliations:** 1 School of Management, ESSCA, Lyon, France; 2 School of Economics, The University of Queensland, St Lucia, Australia

**Keywords:** Risk, Imitation, Social pressure, Peer effects

## Abstract

We report the results of an experiment on how individual risk taking clusters together when subjects are informed of peers’ previous risk taking decisions. Subjects are asked how much of their endowment they wish to allocate in a lottery in which there is a 50% chance the amount they invest will be tripled and a 50% chance their investment will be lost. We use a 2 × 2 factorial design varying: (i) whether the subjects initially observed high or low investment social anchors, (ii) whether information about the investment decisions of other subjects in their social group is provided. We find strong evidence that individuals' risk taking decisions are malleable to that of their peers, which in turn leads to social clustering of risk taking. Social anchors shape initial risk taking, with mean investment then converging to a high level across treatments.

**Supplementary Information:**

The online version contains supplementary material available at 10.1007/s11238-023-09927-x.

## Introduction

Many natural world examples suggest social interdependencies in risk taking, e.g., in the context of the development of different cultures of entrepreneurship (Çelikkol et al., 2019), farming decisions (Fafchamps et al., 2015), stock market bubbles and crashes (Shiller, 1984), panic risk buying at the early stages of the COVID-19 pandemic (Sim et al. 2020), or face mask adoption in COVID-19 pandemic hotspots (Denworth 2020). A growing literature shows that agents’ propensity to take risks is socially embedded.^1^ That is, it depends on the risk taking decisions of others. Such behavioral imitation patterns exist independently of additional institutional features of markets that may enhance the extent to which positively correlated risk taking behavior may take place. For example, in a stock market environment, such institutional features include ‘riding the bubble’ speculative activities (Moinas & Pouget, 2013), social chat (e.g., Mizrach & Weerts, 2009), groupthink (e.g., Bénabou, 2013), and confusion (e.g., Hargreaves Heap & Zizzo, 2011).

A key implication of the social embeddedness of agents’ propensity to take risks is that risk taking decisions should be more clustered together than if agents did not rely on others’ risk taking decisions. However, this implication has not been tested. The main contribution of this paper is to address this gap. Specifically, our experiment is innovative in showing in a general setting that peer effects in risk taking lead to social clustering in individuals’ investment, even in very minimal settings like ours. That is, when agents are placed in groups, their investment levels tend to cluster together relative to an environment where there are no groups. There is, therefore, evidence of endogenous emergence of different social cultures of risk taking that are group specific, even in a stylized environment where there is no financial reward or explicit social reward for this to occur.

We are able to show social clustering of investment allowing for very different average initial investment levels. We induce these by employing initial social anchors, defined as information about peers’ risk taking decisions from a past session and provided at the beginning of the experiment—to shape risk taking decisions. Our results suggest that initial social anchors have significant effects: on average subjects in the high anchor treatments invested twice as much relative to subjects in the low anchor treatments. However, the role of initial social anchors diminishes with time, with average investment converging to high investment levels, albeit with significant between-group heterogeneity.

The rest of this brief report is structured as follows. Section 2 presents the experimental design and hypotheses. Section 3 reports our results, and Section 4 discusses our findings and concludes. In the online appendix, we provide the instructions, and additional econometric analysis to check the robustness of our results.

## Experimental design

### Treatments

In all treatments, participants engage in ten periods of the Gneezy and Potters (1997) (henceforth, GP) risk elicitation task. At the beginning of each period, each subject receives 90 ECU’s and has to decide how much they wish to allocate in an asset in which there is a 50% chance the amount they invest will be tripled and a 50% chance their investment will be lost. We use a 2 × 2 factorial design in which we vary, between subjects, (i) whether the participants observe *Low* or *High* initial social anchors in the first period of the experiment, and (ii) whether social information is provided from the second period onwards (Table 1).

**Table 1:** Treatments

	Social anchor	
	Low	High
Social Information		
With	L	H
Without	LNSI	HNSI

L and H stand for Low and High respectively, while NSI stands for No Social Information.

In the *low anchor treatment*s (i.e., in L and LNSI), in the first period and before their initial investment, the participants are informed that: “In a previous session other participants invested 10, 10, 20, and 25 ECU”. Similarly, in the *high anchor treatments* they observe the same message but with the values 75, 80, 90 and 90. The statements were truthful: as for example in Yoon and Fong (2019), the initial social anchors were selected from genuine investment decisions observed in a preliminary treatment, conducted with the objective to collect initial social anchors for our experiment.

Investment could range between 0 and 100. We selected low vs. high initial social anchors to help clearly identify the anchoring effect that we are trying to capture. The initial social anchors were perfectly symmetric around the mid-point investment of 50 (with a mean of 16.25 and 83.75 respectively) and otherwise having precisely the same distribution (with a common standard deviation of 7.5). This helped us control for any potential distributional confounds that could otherwise explain in the effects of the initial social anchors.

**Fig. 1:**
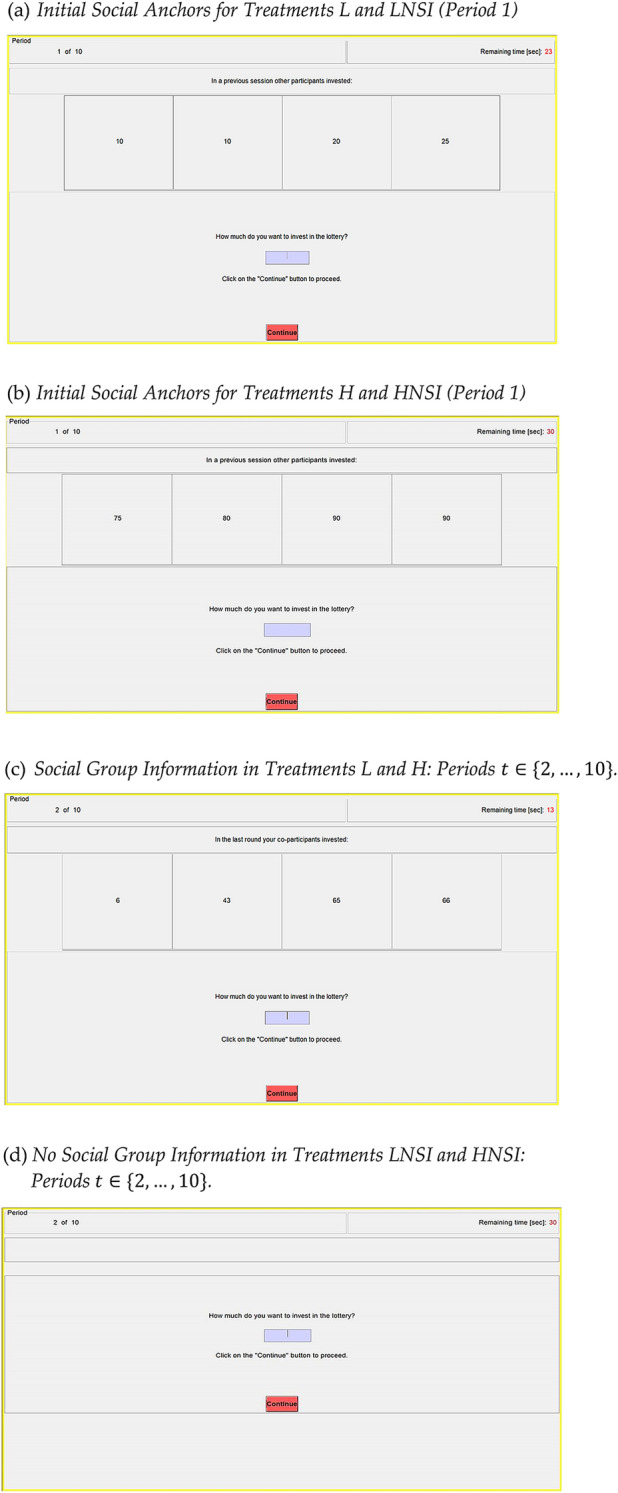
Initial social anchors and sample social information

In the treatments *with social information* (i.e., in L and H), subjects were assigned in groups of five and from period 2 onwards, before they invested, they were informed about how much each other participant in their group invested in the previous period. The groups were fixed throughout the experiment, and this was common knowledge. In the treatments *without social information* (i.e., in LNSI and HNSI), subjects were not assigned in groups, and they were not provided with social information regarding the investment decisions of other participants. Figure 1 shows how the initial social anchors in period 1 and (sample) social information from period 2 were provided to subjects in terms of computer screenshots.

### Hypotheses

Our experimental treatments tie to the following hypotheses.

**Hypothesis 1 (H1):** *Investment rates in H and HNSI will be higher than the investment rates in L and LNSI in the first period*.

**Hypothesis 2 (H2):** *Investment in L and H in period t will be a positive function of peer investment in period t–1*.

As noted in the previous sub-section, we did not have groups in LNSI and HNSI. LNSI and HNSI can nonetheless work as suitable control treatments for the effect of social clustering that we wish to identify in L and H. Specifically, for LNSI and HNSI, we can randomly generate *artificial groups* from the participants in these treatments.^2^ To generate such artificial groups, in the data analysis we randomly chose and put together five subjects who made choices in LNSI and HNSI, even though they were not actually matched together in the experiment itself. We randomly assigned the 60 participants in LNSI and HNSI respectively into 12 groups of 5. This process was repeated 84 times with the same 60 participants to provide 1008 randomly artificial groups per treatment.

We can then verify the standard deviations (SDs) of investment within these artificial groups, and compare them to the SDs of investment in L and H. If social information does not induce social clustering, we would expect the same degree of dispersion (SD) in investment in the real groups of L and H relative to the artificial groups of LNSI and HNSI. If social clustering does take place instead as a result of social information, the SDs of investment within L and H groups will be higher than those in LNSI and HNSI artificial groups. This prediction is encapsulated in H3.

**Hypothesis 3 (H3):** *The within group standard deviations in investment rates in L and H will be lower than the standard deviations observed in artificial groups based on the investment rates observed in LNSI and HNSI*.

Note that H3 is phrased in terms of L and H relative to control treatments, as opposed to being about what happens between the first period and the last period in L and H. The reason is that there may be general time trend effects from repeated play and learning that may confound the effect of social clustering of investment if we compare period 1 and 10 within the same treatment. Instead, by comparing within group standard deviations in L and H with those in artificial groups in LNSI and HNSI, we can control for such effects and purely identify whether there is social clustering as a result of social feedback.

### Procedures

The experiment was conducted at the University of Queensland Behavioural and Economic Science Cluster between March and July 2019. It was programmed in z-Tree (Fischbacher, 2007) and lasted around 40 min. In addition to the decisions from the GP task, at the end of the experiment all subjects completed a standard 16-item variant of the social desirability scale (Stöber, 2001), which we use as an independent measure of subjects’ sensitivity to social pressure (see Zizzo & Fleming, 2011) and correspondingly as a control for potential experimenter demand effects (Zizzo, 2010). We also collected demographic information.

Subjects were paid for one of ten periods selected at random at the end of the experiment to minimize wealth and hedging effects. The average earnings were AUD 19.02 (approximately USD 13.50 at the time).

260 university students participated in our experiment (70 in L and H, and 60 in LNSI and HNSI) of which 91% were undergraduate students, 55% were female, 50% spoke English as their native language and 75% reported economics as their main field of study. Our experiment relies on the random allocation of subjects to treatments, and we find that individual characteristics were not statistically different across treatments, showing that randomization was effective (see section A2.2 of online appendix). The number of subjects for each treatment was determined based on effect sizes in previous related research and in particular Celse et al. (2021) and Rohde et al. (2011).

## Results

### Social anchors, information and mean investment

**Fig. 2:**
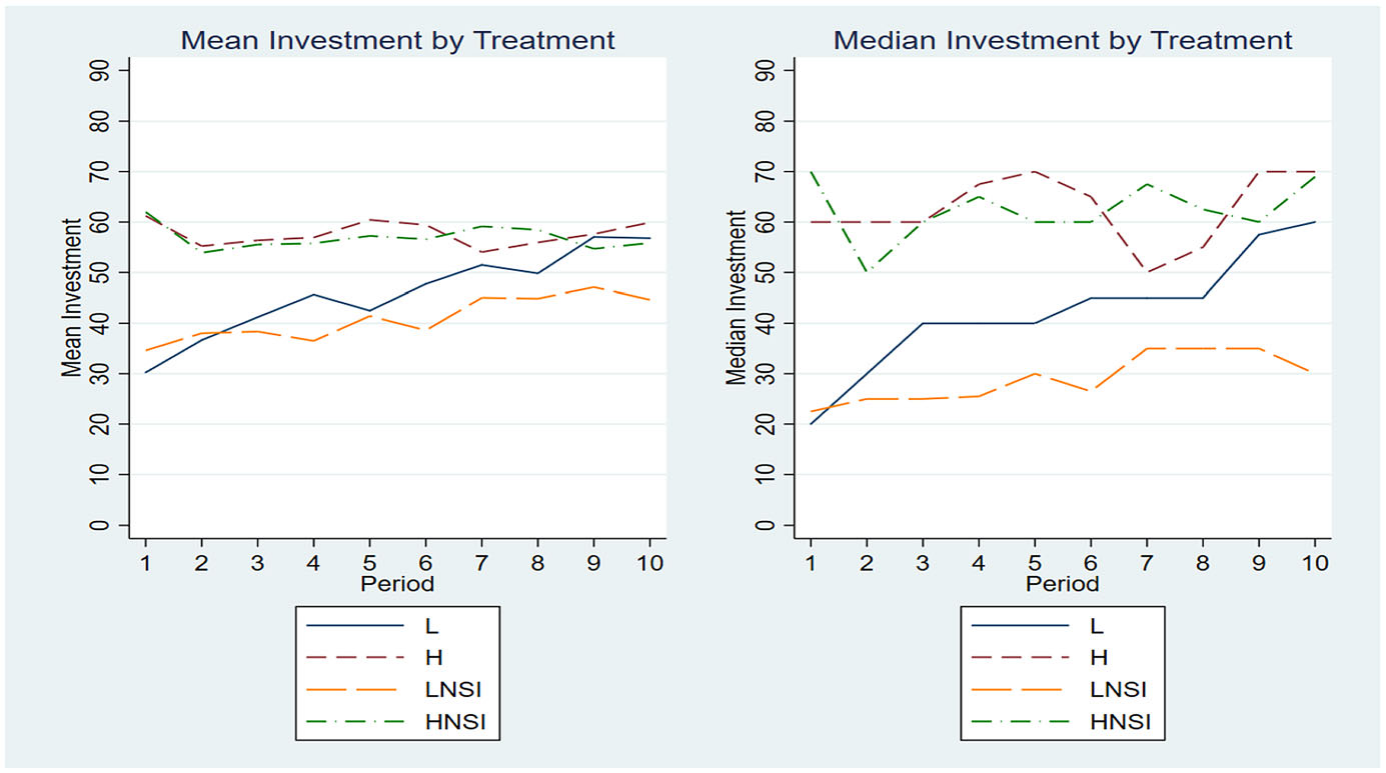
Mean and median investment by period

Figure 2 shows the mean and median investments over time for each of our treatments. Our social anchors successfully induce significant average differences in the risk taking of our subjects in the first period of the experiment.

Specifically, in the first period and consistently with H1, the mean investment levels in H (61.21) and HNSI (61.98) are approximately twice as large relative to L (30.24) and LNSI (34.62) and statistically significantly different from each other (Mann–Whitney test, *p* < 0.001). Similarly, the median is three times higher in H (60.61) and HNSI (69.98) relative to L (20.01) and LNSI (22.86) (Mann–Whitney test, *p* < 0.001). Furthermore, the mean investment levels across all periods in HNSI and in LNSI are 56.91 and 40.92, respectively (Mann–Whitney test, *p* < 0.001), suggesting that initial social anchors lead to long-term effects in investment levels in the absence of social information.

To test H1, Table 2 reports Tobit regressions on investment in period 1. Model 1 includes only our treatment variables with H as baseline. Model 2 includes controls for individual characteristics.^3^ The regression results are in line with Fig. 1 and with H1. As a robustness check, they are further corroborated by random effects panel Tobit regressions provided in an online appendix.

**Table 2:** Tobit regressions on investment in period 1

	Model 1	Model 2
L	– 13.35*** (5.179)	– 34.65*** (4.806)
LNSI	– 17.98*** (2.152)	– 27.46*** (4.981)
HNSI	– 1.693 (2.158)	0.519 (5.019)
Controls	No	Yes
Constant	54.31** (3.780)	67.99** (8.421)
*N*	260	260
*χ*^*2*^	96.69	78.69

Investment is censored at 0 and 90

∗∗∗, ∗∗, ∗ indicate significance at the 0.1%, 1%, and 5% level, respectively. Our control variables are the following: gender, English as a first language, current or prior study of Economics and their average score on Stöber’s (2001) social desirability scale

**Result 1:** *Subjects who observed the high social anchors (i.e. H and HNSI), invested more than the subjects who observed the low social anchors in the first period (i.e. L and LNSI).*

### Social interdependence in investment

To verify that social group information affects investment decisions, we conducted three random effects Tobit regressions on investment in time $t$ focusing on the data from H and L treatments, censoring investment at 0 and 90 (Table 3). Model 1 uses H as our baseline treatment and decomposes the time trend observed in L (but not in H) using interaction variables. The results in Model 1 are similar to the ones observed in Model 2 of Table 2. In Model 2, we introduce *Others’ Investment in *$t-1$*,* which captures the average investment of the other subjects in the group in the previous round. Others’ Investment in $t-1$ is highly statistically significant, implying social interdependence of investment levels within groups in accordance with H2. Model 3 finds this robust to controlling for individual characteristics.

**Table 3:** Random effects Tobit regressions on investment rates over time in L & H

	Model 1	Model 2	Model 3
L	– 29.253*** (5.298)	– 17.382*** (6.35)	– 18.452*** (6.310)
L*Period	3.408*** (0.359)	2.466*** (0.471)	2.463*** (0.471)
H*Period	0.341 (0.362)	0.776 (0.43)	0.776 (0.430)
Others’ Investment* in* $t-1$		0.246** (0.074)	0.248** (0.074)
Controls	No	No	Yes
Constant	60.24*** (3.755)	43.43*** (6.085)	41.06*** (11.29)
*N*	1400	1260	1260
Wald* χ*^*2*^	98.82	73.89	80.70

Investment is censored at 0 and 90. ∗  ∗  ∗ , ∗  ∗ , ∗ indicate significance at the 0.1%, 1%, and 5% level, respectively. Our control variables are the following: gender, English as a first language, current or prior study of Economics and their average score on Stöber’s (2001) social desirability scale. In model 1 investment is censored at 0 in 47 observations. And at 90 at 317 observations. In models 2 and 3, investment is censored at 0 in 44 observations and at 90 in 300 observations. Simple OLS are reported in table A2.8 in the online appendix yielding qualitatively similar results.

**Result 2:** *Investment in L and H in period t is a positive function of peer investment in period t-1.*

### Social clustering of investment decisions

H3 follows from the social interdependence of investment predicted by H2. According to H3, the standard deviations (SDs) of investment in groups with information (as in L and H) should be lower than the investment in comparable groups without information. As discussed in Sect. 2.2, a suitable control for the within-group SDs in L and H is provided by the within-group SDs of randomly generated artificial groups drawn from LNSI and HNSI.

**Fig. 3:**
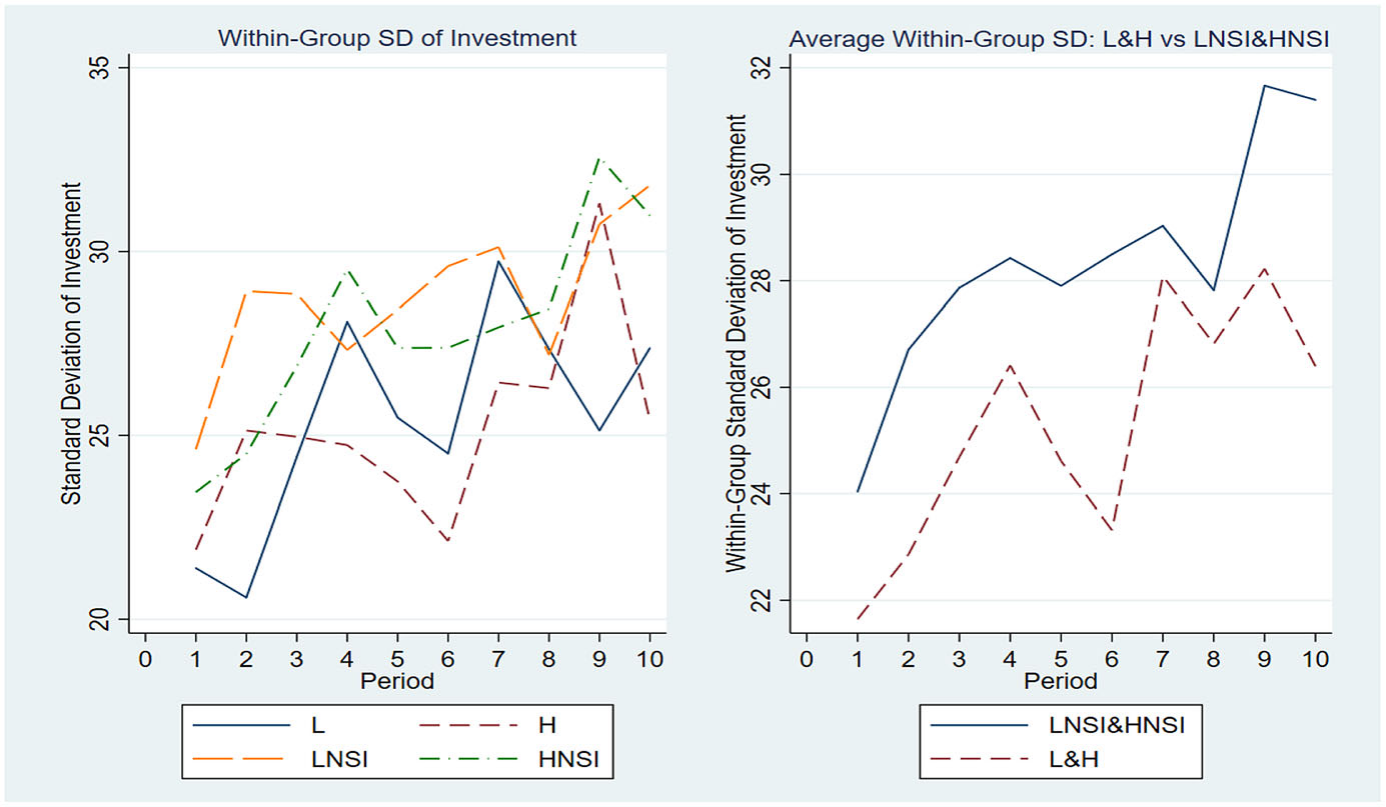
Within-group standard deviations over time

Figure 3 displays how mean SDs change with time. In all treatments, SDs increase over time, suggesting a progressively lower pull of initial social anchors on investment. However, Fig. 3 suggests that the effect is less pronounced in L than in LNSI, and in H than in HNSI. Specifically, within-group SDs are lower when social information is provided as predicted by H3 (Mann–Whitney test: SD L & H relative to HNSI & LNSI, *p*-value = 0.01), as shown in the right panel of Fig. 3.^4^

To further test H3, Table 4 reports panel regressions on the standard deviation of group investment with robust standard errors. Model 1 presents the simplest specification, using the treatments with social group information (i.e. L & H) as the baseline, and introducing a dummy variable (LNSI & HNSI) for these two treatments, relying on artificial groups data. In line with H3, in the treatments without social group information the standard deviation of investment is significantly higher. Additionally, in line with Fig. 3, we find a positive time trend. In Model 2, we test whether the increase in standard deviations over time is different between groups with social group information relative to the treatments without social group information. However, we find no such effect, suggesting that social group information has a similar effect on risk taking clustering across treatments over time.

**Table 4:** Panel regressions on standard deviations of group investment with robust standard errors

Panel regressions. Dependent variable: Investment SD Periods:1–10			
	Model 1	Model 2	Model 3
*LNSI & HNSI*	3.028^***^ (0.569)	2.748^***^ (0.733)	2.757^***^ (0.735)
*Period*	0.624^***^ (0.00977)	0.574^***^ (0.0771)	0.574^***^ (0.0772)
LSNI & HNSI*Period		0.0508 (0.0778)	0.0508 (0.0778)
*Controls*	No	No	Yes
*Constant*	21.87^***^ (0.568)	22.15^***^ (0.727)	23.74^***^ (0.778)
*# Observations*	102,200	102,200	102,200
*R*^*2*^	0.0395	0.0395	0.0427

***Indicates significance at the 0.1% level. In L and H, there are 700 within-group standard deviation observations each, and for LNSI and HNSI there are 50,400 observations drawn from the artificial groups for each treatment. For treatments L and H, the 700 observations are a function of the number of participants in each treatment (*n* = 70) and the number of periods they invested in (*t* = 10). For treatments LNSI and HNSI, the number of observations is determined by the total number of simulated participants (*n* = 60 × 84 = 5,040) and the number of periods they invested in (*t* = 10). LNSI & HNSI Is a dummy variable taking the value 1 in the LNSI and HNSI treatments and 0 otherwise. Our control variables are the following: gender, English as a first language, current or prior study of Economics and their average score on Stöber’s (2001) social desirability scale.

**Result 3:** *Investment rates cluster by social groups.*

It might be seen puzzling that the mean standard deviations start lower in the first period in both treatments with social information. However, a plausible explanation is that, as subjects in the treatments with social information are aware that they will later receive feedback about their peers, they may adopt a more socially prudent attitude that lowers investment heterogeneity already in the first period.

## Conclusion

We conducted an economic experiment that tests for social interdependence in individual risk taking decisions. Even in our minimal setting, which abstracts from institutional features of the decision environment (e.g. in financial markets), and where the expected return of the asset is known and fixed, we find clear evidence of social interdependence of risk taking decisions at the group level, leading to social clustering of risk taking decisions.

The apparent process of discovery of a common mean acceptable level of investment at the treatment level over time obscures the enormous heterogeneity in group dynamics and the clustering of investment decisions at the group level. In this sense, our results appear consistent with the idea that, rather than subjects simply discovering their intrinsic risk preferences over time, their preferences are shaped at least to some degree by the group interaction.^5^ Hedging is not a possible explanation of our findings either, since subjects knew that they were being paid only for one out of the ten investment decisions, selected at random at the end of the experiment.

Our results are robust to large differences in average initial investment, which we induce using initial social anchors. Our results suggest that initial social anchors can have significant effects on investment decisions, especially in the absence of information regarding peer investment, although the effect does appear to reduce over time, with mean convergence to high investment levels.^6^ While mean social investment remained stable in our H treatment, it increased considerably with time in L. Our analysis of investment dynamics suggests that this is not due to agents responding to social information differently. Instead, our interpretation of this observation is that, given subjects’ tolerance to risk, when the initial social anchors are low, any drift toward higher investment (as observed in LNSI) is enhanced by a social race to catch up with those who are investing relatively more and likely to be earning more (see Fafchamps et al., 2015; Feltovich and Ejebu 2014; Müller & Rau, 2019; Gill et al. 2018). This speaks toward competitive preferences rather than imitation being the driver of why information about the others’ investment matters in shaping risk tolerance.

One potential concern with social anchors is that they are a source of experimenter demand effects (Zizzo, 2010). However, our results are robust to our measure of social desirability (Stöber, 2001), which is among our control variables and provides a useful control for such effects (Fleming & Zizzo, 2015). In addition, if experimental anchors were a source of behavioral change due to experimenter demand effects, we should observe that they always matter; instead, they have been found to matter only where they are plausible and relevant (Li et al., 2021; Sugden et al., 2013), as in our case. One dimension we do not consider in our experiment is the specific psychological mechanism behind social interdependence. As investment in the asset is positively correlated with expected payoffs, social interdependence in investment behavior may be driven either by a genuine motivation to imitate the actions of others (e.g., Cooper & Rege 2011; Lahno & Serra-Garcia, 2015) or alternatively by outcome-based preferences models, such as competitive preferences in outcomes (e.g., Brenner, 1987; Bault et al., 2008; Hillebrandt and Steinhorth 2020). Our experiment was not designed to answer this question, and as such we cannot provide a conclusive answer on the psychological mechanism that drives the observed behavior. However, as noted above, the mean convergence to a high investment level provides suggestive evidence in support of competitive preferences. This insight is consistent with Celse et al. (2021), who run a horse race among different models that may explain socially interdependent risk taking in Gneezy and Potters (1997) investment tasks. They consider this question by providing both information regarding investment decisions and investment outcomes of a peer, albeit in a static context. Their results suggest that models of competitive preferences are better suited in explaining interdependence of investment decisions, relative to models of imitation or inequality aversion. However, that study focuses on risk taking decisions in one-shot interactions with social information about one other participant, while here we look at potential social clustering in dynamic settings. Extending our setting by providing only investment outcomes by others, or a combination of investment choices and outcomes, could be an exciting direction for future research. An alternative direction for future research would be to explore how ambiguity over the expected return of the asset would influence risk taking social interdependence.

## Supplementary Information

Below is the link to the electronic supplementary material.

Supplementary file1 (DOCX 1042 KB)

## Footnotes

1 See Boles and Messick (1995), Zizzo (2001), Bault et al. (2008), Delgado et al. (2008), Haisley et al. (2008), Hill and Buss (2010), Cooper and Rege (2011), Rohde and Rohde (2011), Viscusi et al. (2011), Linde and Sonnemans (2012), Bursztyn et al. (2014), Dijk et al. (2014), Friedl et al. (2014), Kuziemko et al. (2014), Mishra et al., (2014, 2015), Fafchamps et al. (2015), Lahno and Serra-Garcia (2015), Schwerter (2015), Brookins et al. (2016), Chao et al. (2017), Gamba et al. (2017), Gioia (2017), Xie et al. (2017), Gantner and Kerschbamer (2018), Lopera and Marchand (2018), Mitton et al. (2018), Gortner and van der Weele (2019), Müller and Rau (2019), Schmidt et al. (2019), Hillenbrandt and Steinorth (2020), and Celse et al. (2021).

2 For clarity, artificial groups are based on actual experimental data, collected as described below. A more complete label would be artificially matched groups, for it is the matching of participants into groups that is artificial; we use the terminology artificial group for brevity.

3 Our control variables in the regressions reported in this paper are the following: gender, English as a first/native language, current or prior study of Economics and their average score on Stöber’s (2001) social desirability scale. This information was collected at the end of the experiment.

4 To conduct the Mann–Whitney test we have taken the average from all periods. As there are 1008 simulated groups in LNSI & HNSI, respectively, and 7 groups in L and H, respectively, the test involves a total of 2030 observations. It might be seen puzzling that the mean standard deviations start lower in the first period in both treatments with social information. However, we expect that, as subjects in the treatments with social information are aware that they will later receive feedback about their peers, they may adopt a more socially prudent attitude that lowers investment heterogeneity already in the first period.

5 There is a related traditional debate on whether preferences are ‘discovered’ or shaped as agents engaged in repeated decisions (e.g., Cubitt et al., 2001; Isoni et al., 2016).

6 This is consistent with research from other settings on the effectiveness of anchors that are perceived relevant (e.g., Li et al., 2021; Sugden et al., 2013), where social anchors are seen as relevant.
