# Supplementary material for: Socially interdependent risk taking
Source: Theory Decis. 2023 Apr 19:1–14. Online ahead of print. doi: 10.1007/s11238-023-09927-x (PMC10116893; doi:10.1007/s11238-023-09927-x)
Supplement: Supplementary file 1 — Supplementary file1 (DOCX 1042 KB) [file 11238_2023_9927_MOESM1_ESM.docx]

# Socially Interdependent Risk Taking

# Online Appendix

Table of Contents

[A1. Experimental Design 2](#_Toc109718658)

[A1.1 Instructions 2](#_Toc109718659)

[A1.2 Quiz Questions (computerized) 3](#_Toc109718660)

[Reasoning for Questions 4](#_Toc109718661)

[A1.3 List of SDS16 Questions (Stober, 2001) 4](#_Toc109718662)

[A1.4 zTree screenshots 5](#_Toc109718663)

[Welcome Screen 5](#_Toc109718664)

[Understanding Question 1 and Answer 6](#_Toc109718665)

[Understanding Question 2 and Answer 7](#_Toc109718666)

[Understanding Question 3 and Answer 8](#_Toc109718667)

[Understanding Question 4 and Answer 9](#_Toc109718668)

[Part 1 10](#_Toc109718669)

[Initial Social Anchors for Treatments L and LNSI (Period 1) 10](#_Toc109718670)

[Initial Social Anchors for Treatments H and HNSI (Period 1) 11](#_Toc109718671)

[Social Group Information in Treatments L and H: Periods $t\in\{2,\ldots, 10\}$. 11](#_Toc109718672)

[No Social Group Information in Treatments LNSI and HNSI: Periods $t\in\{2,\ldots, 10\}$. 12](#_Toc109718673)

[Lottery Outcome After each Period 13](#_Toc109718674)

[Part 2 13](#_Toc109718675)

[Demographic Questions 14](#_Toc109718676)

[Final Results 15](#_Toc109718677)

[A2. Additional Data Analysis 16](#_Toc109718678)

[A2.1 Preliminary treatment summary statistics 16](#_Toc109718679)

[A2.2 Demographic characteristics by treatment 17](#_Toc109718680)

[A2.3 Random effects Tobit regressions on investment rates over time 18](#_Toc109718681)

[A2.4 Frequency of investment by treatment 18](#_Toc109718682)

[A2.5 Multilevel mixed-effects linear regression (Treatments L & H) 20](#_Toc109718683)

[A2.6 Frequencies of within-group standard deviations 21](#_Toc109718684)

[A2.7 Investment change 21](#_Toc109718685)

[A2.8 Explaining investment decisions using OLS regressions 23](#_Toc109718686)

# A1. Experimental Design

## A1.1 Instructions

**General Instructions**

This is an experimental session about decision making. Please read these instructions carefully, as they will help you to understand the nature of the decisions you will be asked to take today. In this session, your earnings will only depend on a combination of luck and the choices you make; they do not depend on the choices made by anyone else**.** Your identity will remain anonymous. You will indicate your decisions directly on the computer. There are neither good nor bad answers.

From now and until the end of the session, we ask you to switch off your mobile devices and remain silent. Should you have any questions, please raise your hand and an experimenter will come to answer your questions privately.

We will read the instructions aloud and ask you to complete a comprehension questionnaire to ensure you correctly understood them. Once all participants have completed the questionnaire, the session will begin.

You will be given a show up fee of $5 for coming today. In addition, you can earn more based on your decisions. We will be using an experimental currency unit called ECU. In the end, the total ECU you have earned during the session will be converted to AUD at the exchange rate of:  **1 ECU = $0.13.** Stage 1 has ten periods. At the end of the session, your potential earnings from one of the ten periods will be randomly selected by the computer and paid to you in cash along with your show up fee.

This session is made of two stages. **Stage 1**, is explained below. **Stage 2**, consist of an end-of-experiment *questionnaire*.

**Stage 1**

A period has the following steps:

1. **L & H:[** The computer will randomly create groups with five participants in each group. **]**
2. You receive an initial endowment of 90 ECU at the beginning of the stage.
3. **L & H:[** After observing the previous investment decisions,**]** you can invest any amount from 0 ECU up to and including 90 ECU in a lottery. Any amount you invest in the lottery will be tripled with a probability of 50% or will be lost with a probability of 50%.
4. The computer then proceeds to the random draw of the lottery and informs you about your income, the amount you invested in the lottery, the amount you obtained from the lottery, and your period earnings.

In the first period, you will also have the opportunity to observe four different investment amounts, decided by participants from a previous group.

**L & H:[**At the beginning of each subsequent period, you will have the opportunity to view the investment amounts the other four *co-participants* in your group made in the previous periods.**]**

Your period earnings are determined according to the formula below:

$$Period Earnings = Initial Endowment - Amount invested in Lottery + Lottery Outcome+Show up Fee$$

After all participants in your group have gone through Stage 1, Stage 2 begins.

## A1.2 Quiz Questions (computerized)

**Question 1.**

What is the range you can invest in the lottery?

1. 0 to 45 ECU
2. 0 to 90 ECU
3. 0 to 155 ECU

Answer: (2)

**Question 2.**

Which of the following statements is correct?

1. Your potential earnings are dependent on the choices your co-participants make and luck.
2. Your potential earnings are only determined from the choices you make and luck.
3. Your potential earnings are dependent on both your choices, the choices of your co-participants and luck.

Answer: (2)

**Question 3.**

Which of the following statements is correct?

1. Any amount you invest in the lottery will be doubled with a probability of 50% or will be lost with a probability of 50%.
2. Any amount you invest in the lottery will be doubled with a probability of 75% or will be lost with a probability of 25%.
3. Any amount you invest in the lottery will be tripled with a probability of 50% or will be lost with a probability of 50%.

Answer: (3)

**Question 4.**

How many periods will be paid out for this experimental session?

1. All periods
2. Only period 1
3. Only one randomly selected period

Answer: (3)

### Reasoning for Questions

Question 1 is provided to ensure participants know the investment range is between 0 and 90 ECU. Question 2 reinforces the crucial detail that their potential earnings are determined by the decisions they make, and not the decisions of their *co-participants.* Thus, reinforcing the lottery is not a group activity. Question 3 tests participants’ understanding of the potential risks and benefits associated with investing in the lottery. Lastly, Question 4 reminds participants that only one randomly selected round will be used to determine final earnings. This question helps participants understand their potential earnings is not a function of multiple rounds, thus controlling for the potential of wealth effects.

## A1.3 List of SDS16 Questions (Stober, 2001)

The SDS16 questionnaire involves participants responding to 16 statements in which they can either answer true or false. Each statement is presented one at a time. The 16 statements are presented below:

1. I sometimes litter

2. I always admit my mistakes openly and face the potential negative consequences

3. In traffic I am always polite and considerate of others

4. I always accept others’ opinions, even when they don’t agree with my own

5. I take out my bad moods on others now and then

6. There has been an occasion when I took advantage of someone else

7. In conversations I always listen attentively and let others finish their sentences

8. I never hesitate to help someone in case of emergency

9. When I have made a promise, I keep it – no ifs, ands or buts

10. I occasionally speak badly of others behind their back

11. I would never live off other people

12. I always stay friendly and courteous with other people, even when I am stressed out

13. During arguments I always stay objective and matter-of-fact

14. There has been at least one occasion when I failed to return an item that I borrowed

15. I always eat a healthy diet

16. Sometimes I only help because I expect something in return

Note that the value inputs for the SDS17 questionnaire are (True = 0, False = 1) for statements 1, 5, 10, 14, 16, and (True = 1, False = 0) for statements 2, 3, 4, 6, 7, 8, 9, 11, 12, 13, 15. The participants’ SDS17 score is given by the sum of these value inputs.

## A1.4 zTree screenshots

### Welcome Screen


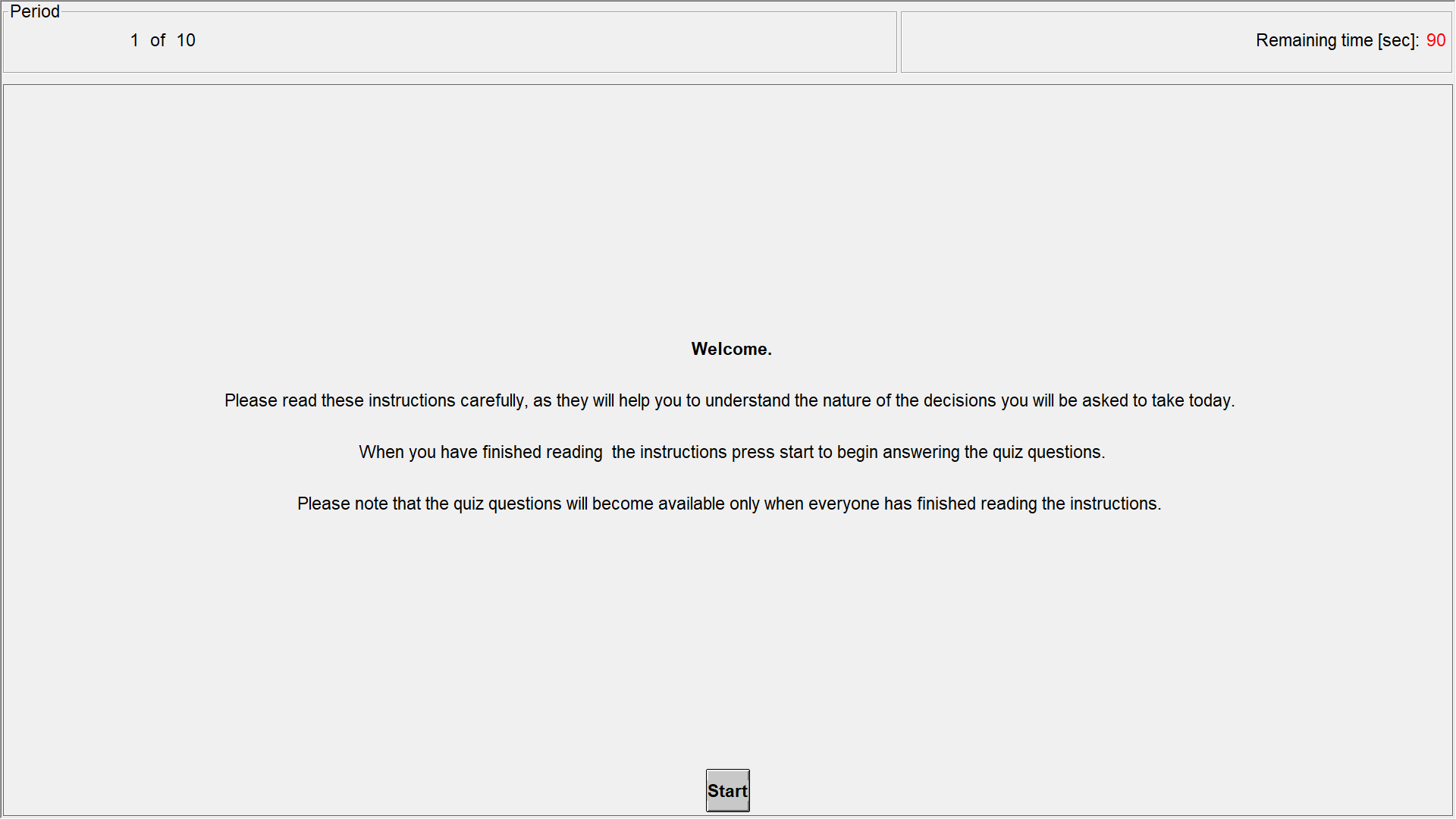


### Understanding Question 1 and Answer


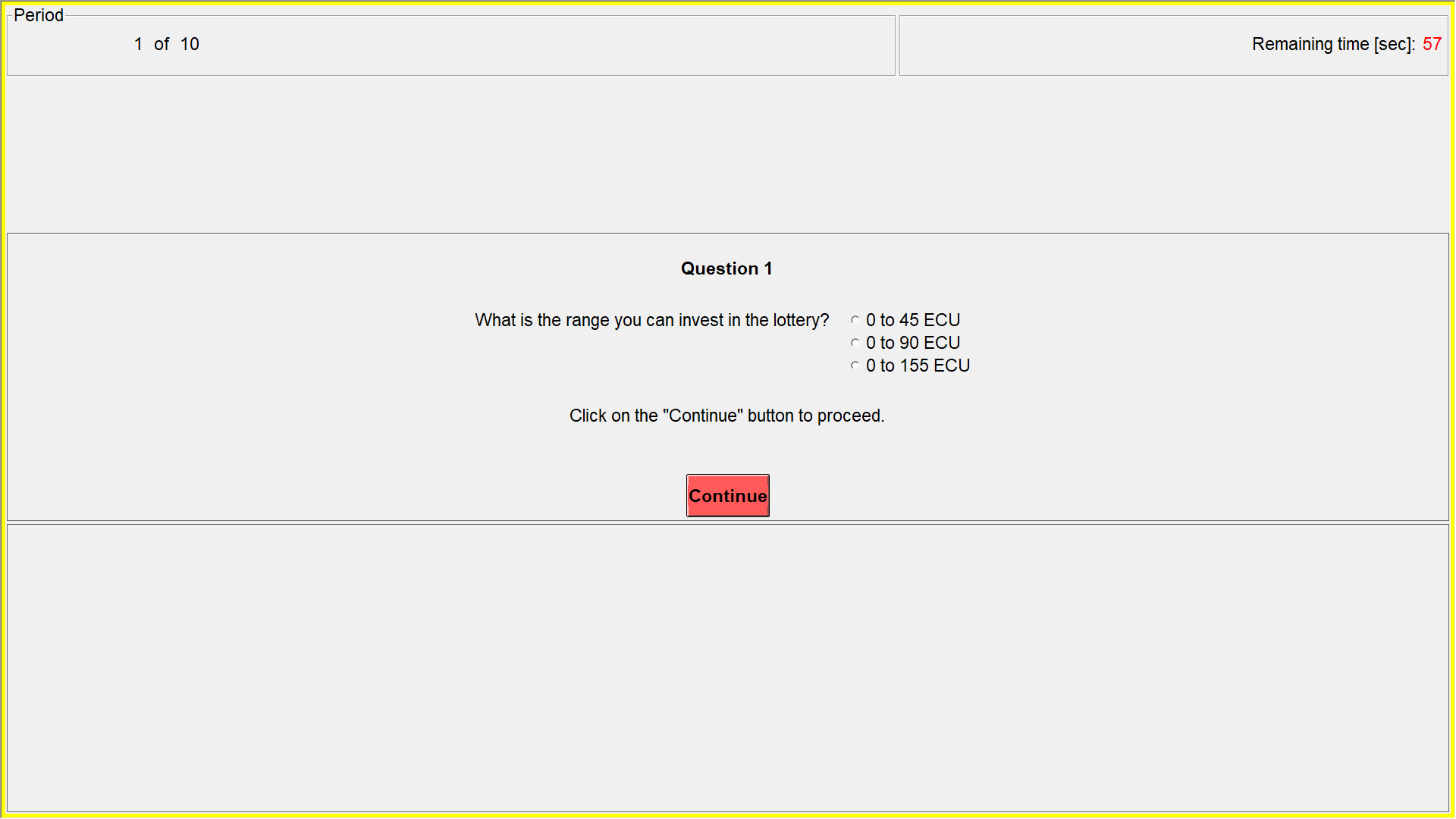


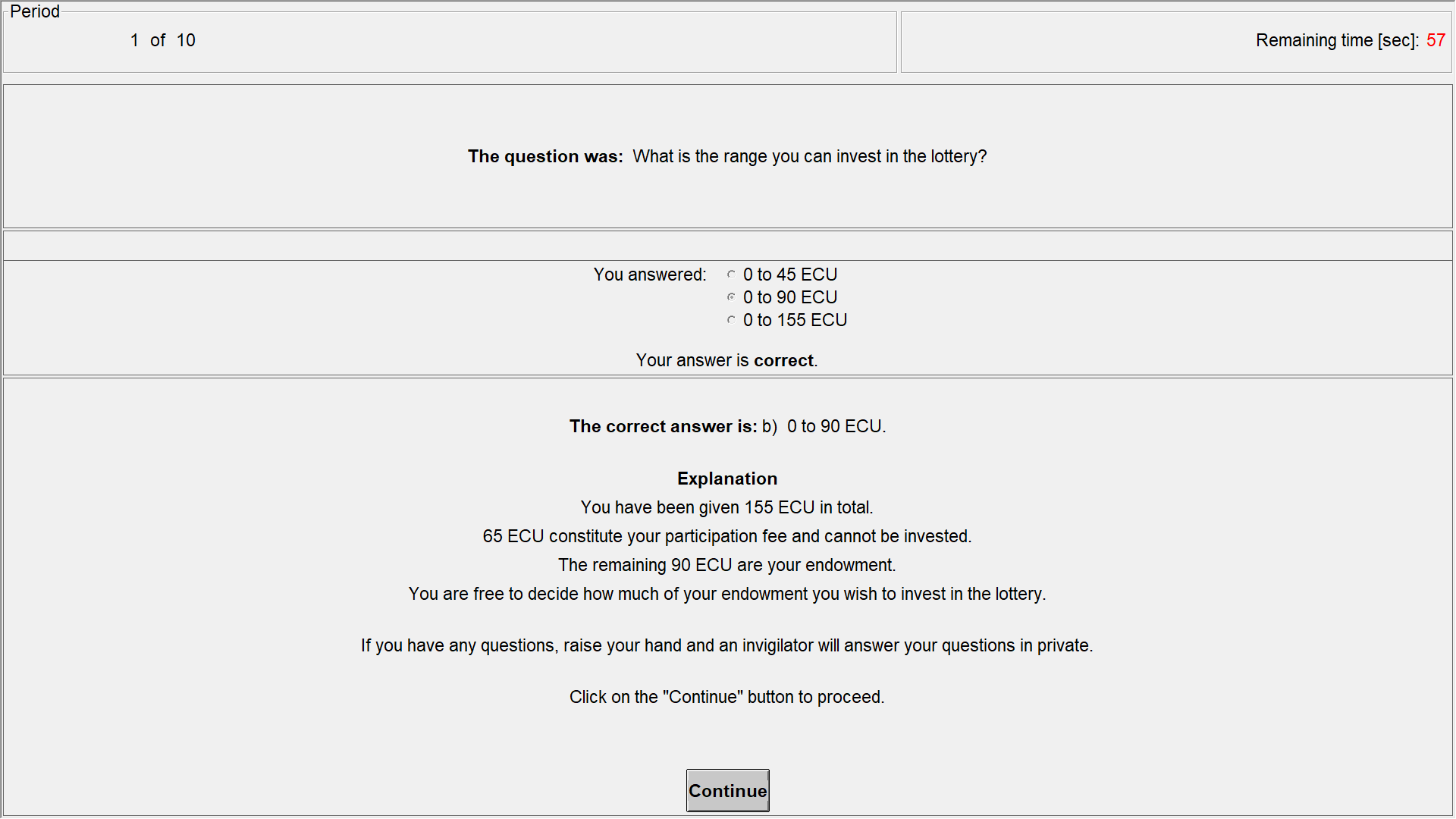


### Understanding Question 2 and Answer


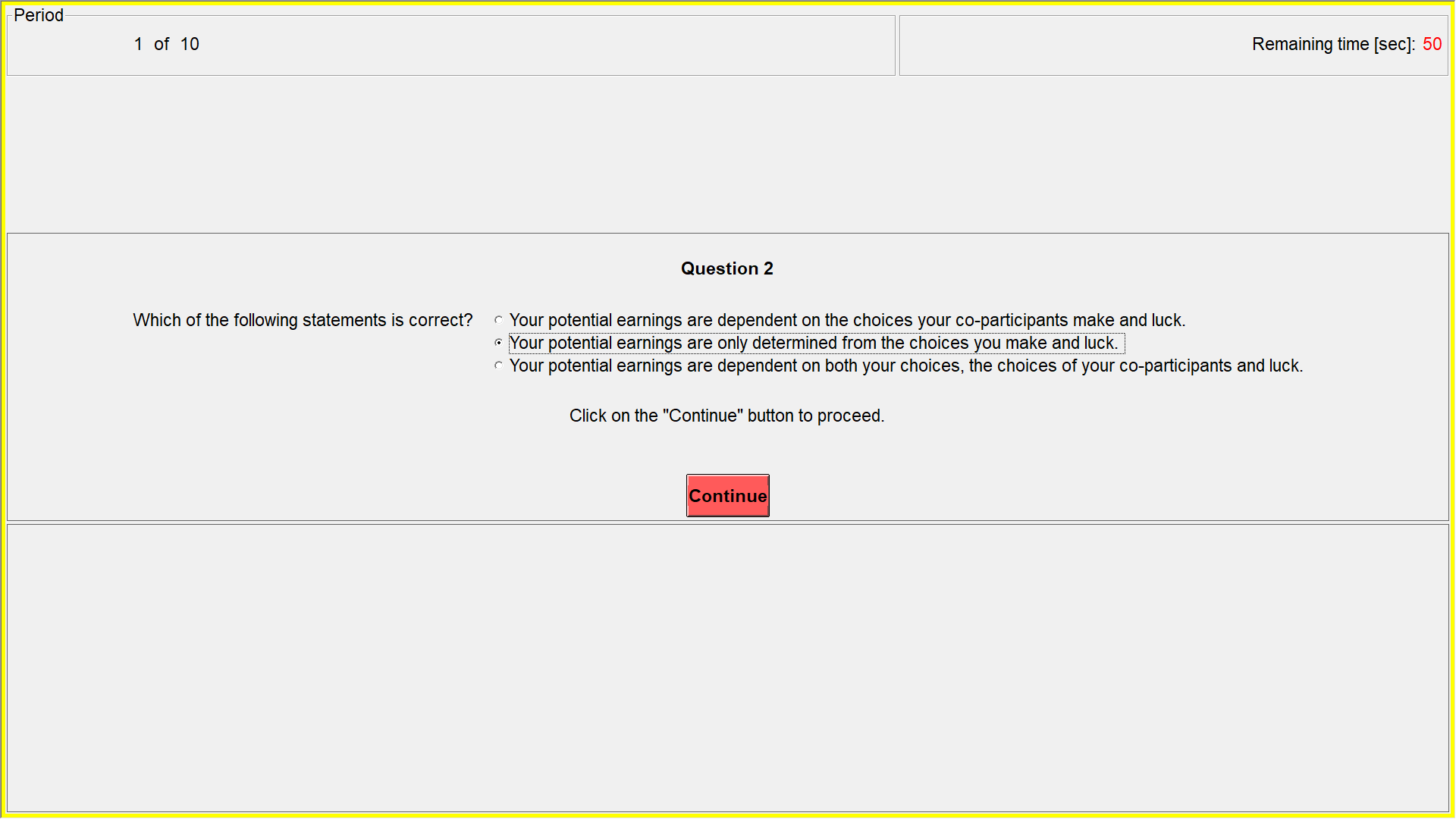


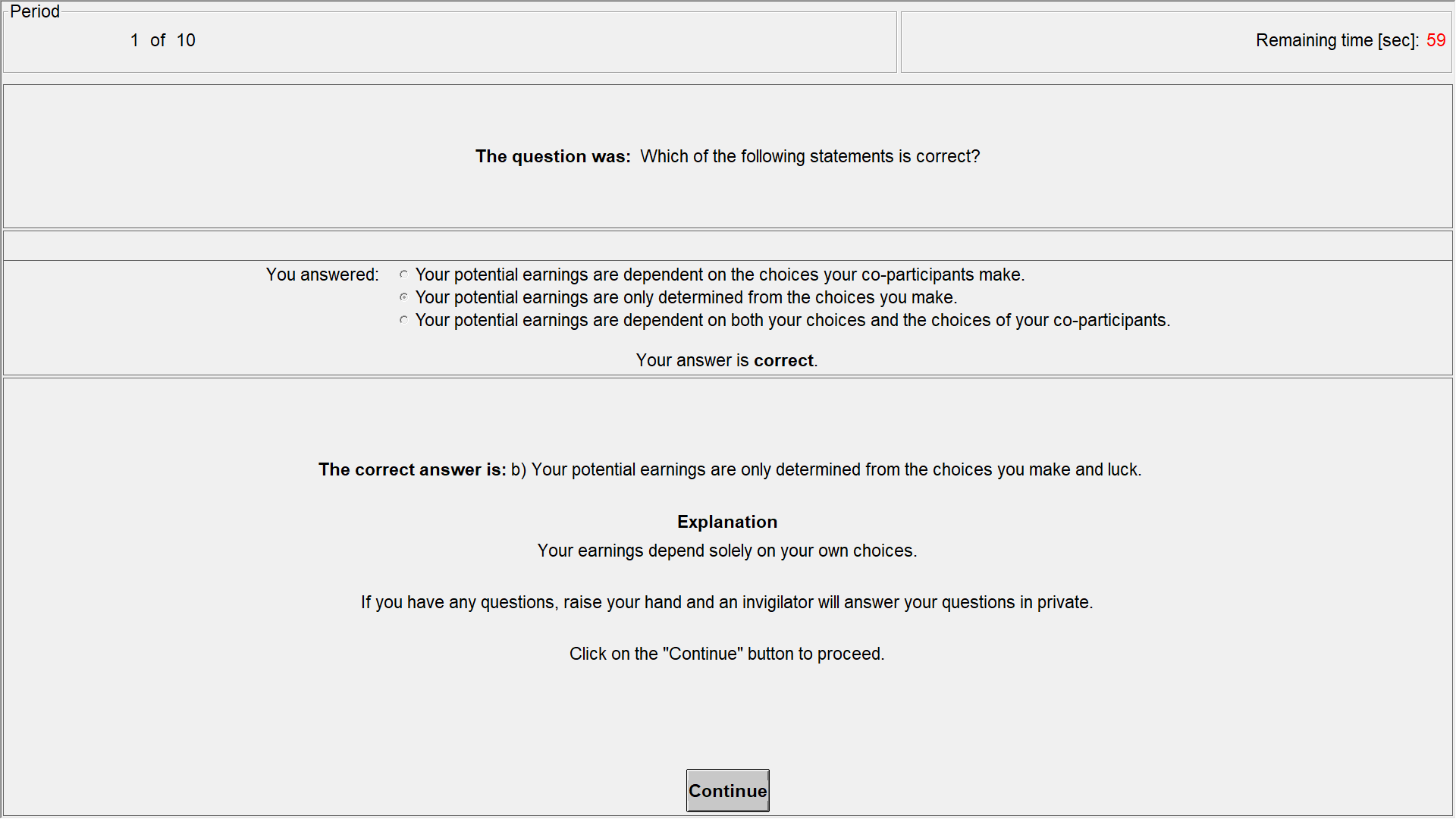


### Understanding Question 3 and Answer


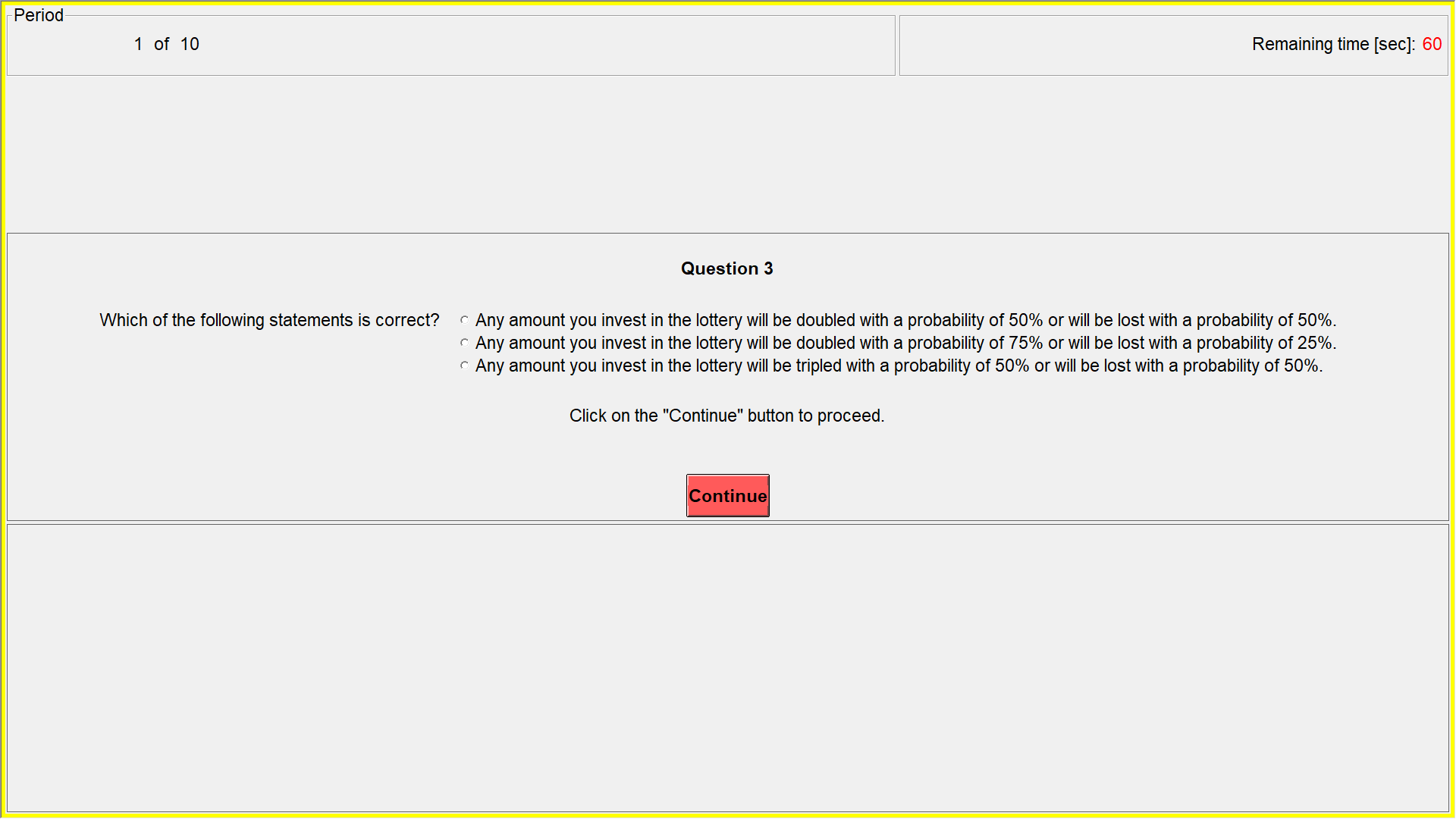


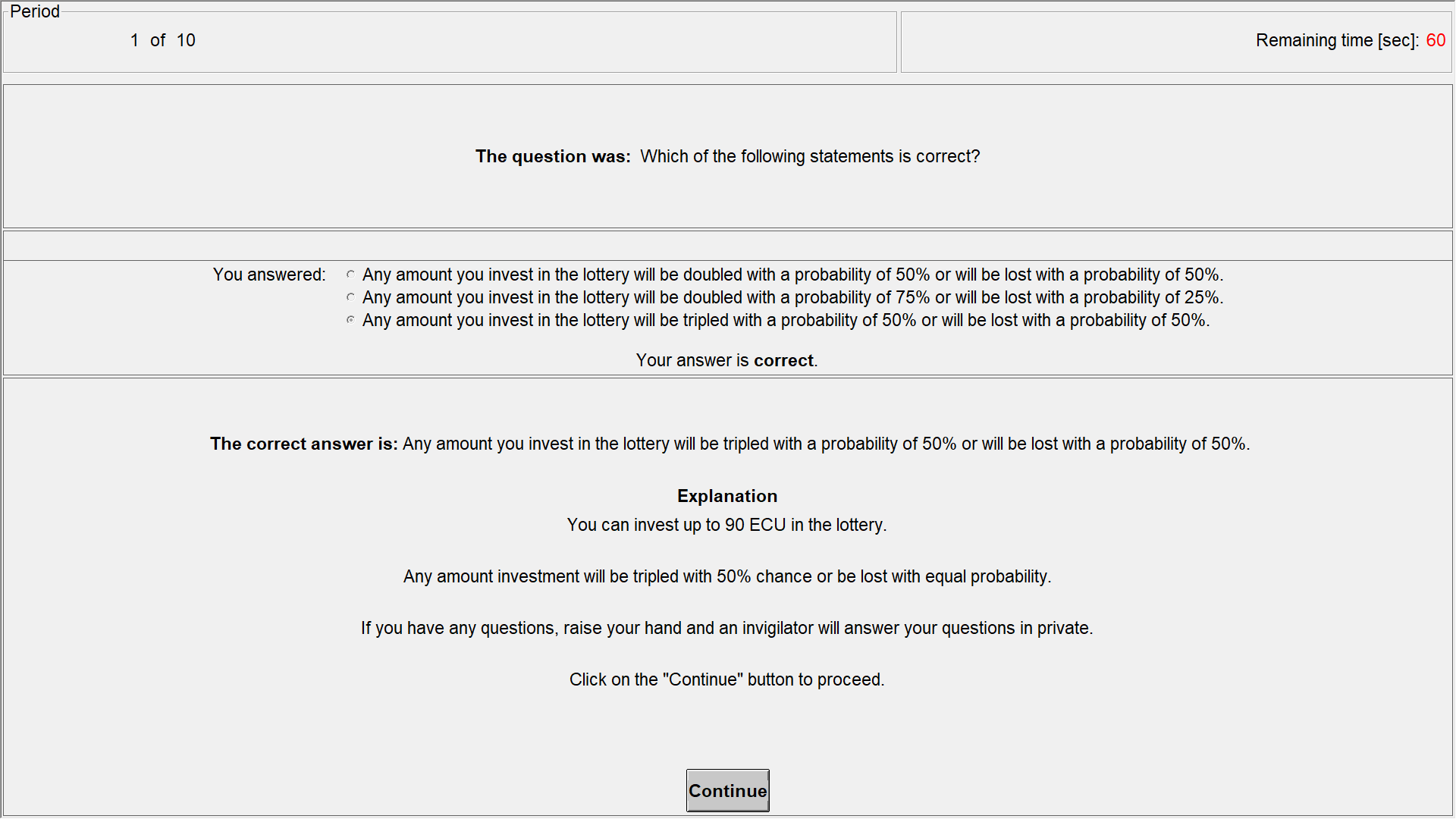


### Understanding Question 4 and Answer


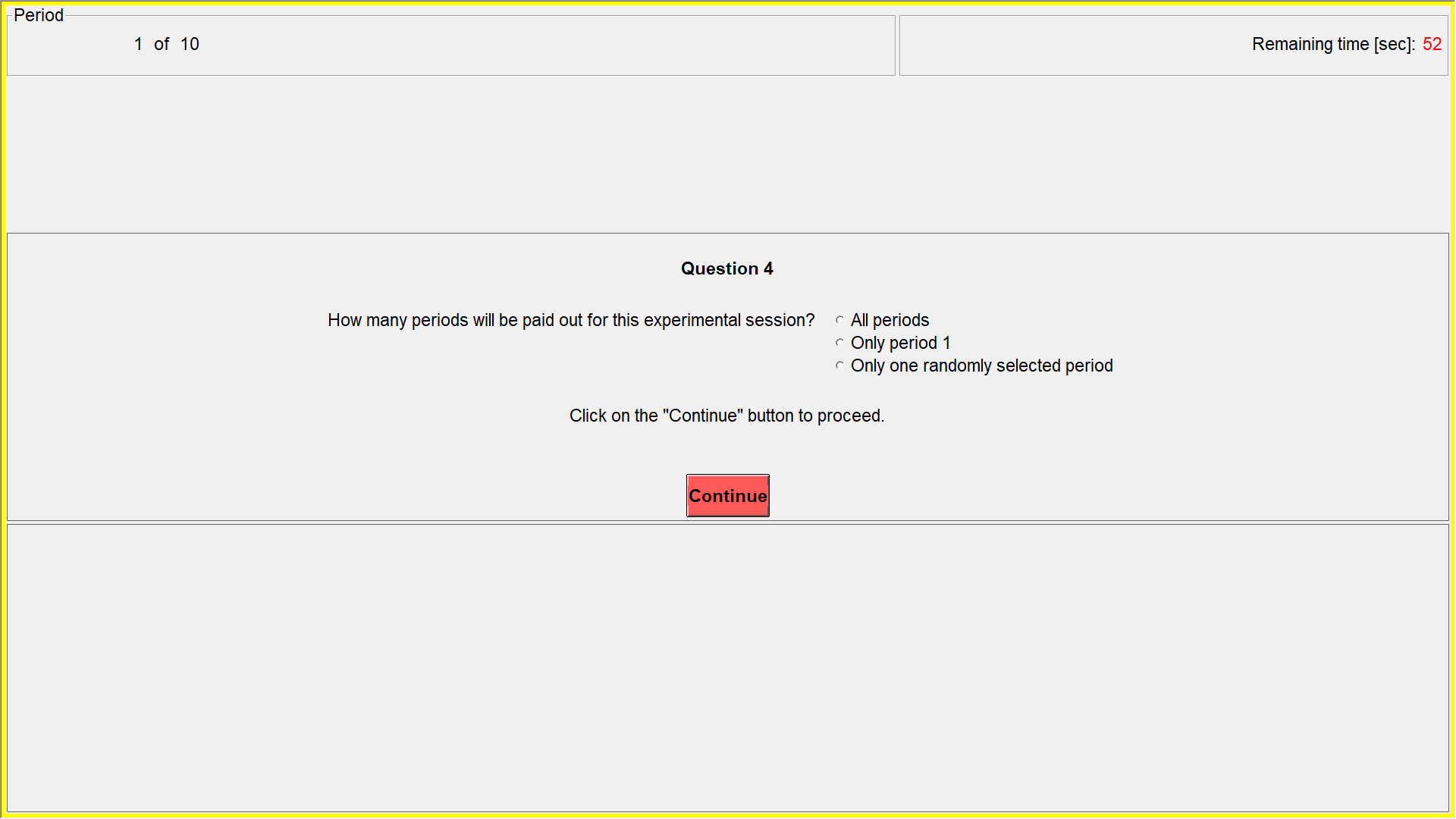


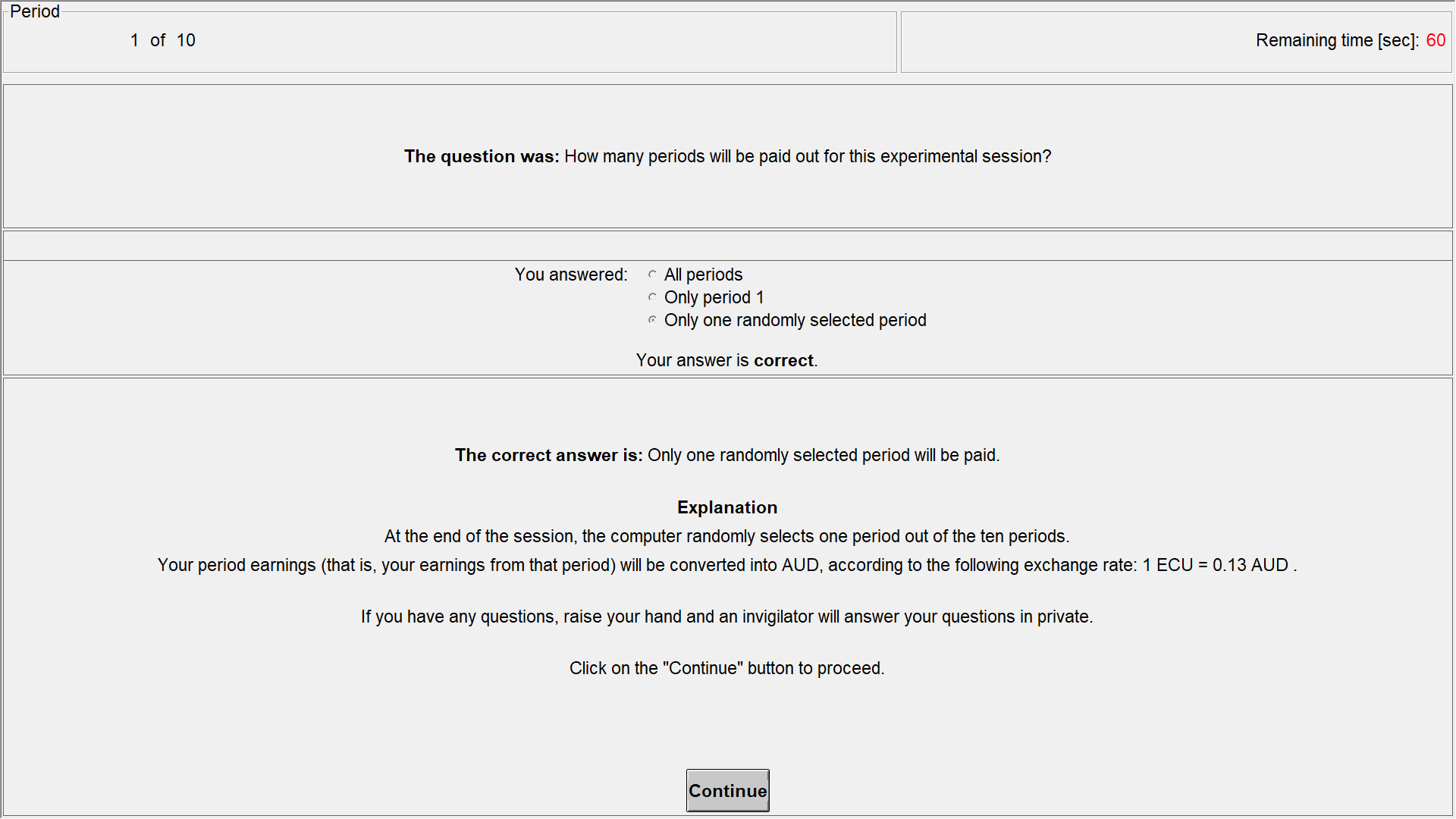


### Part 1


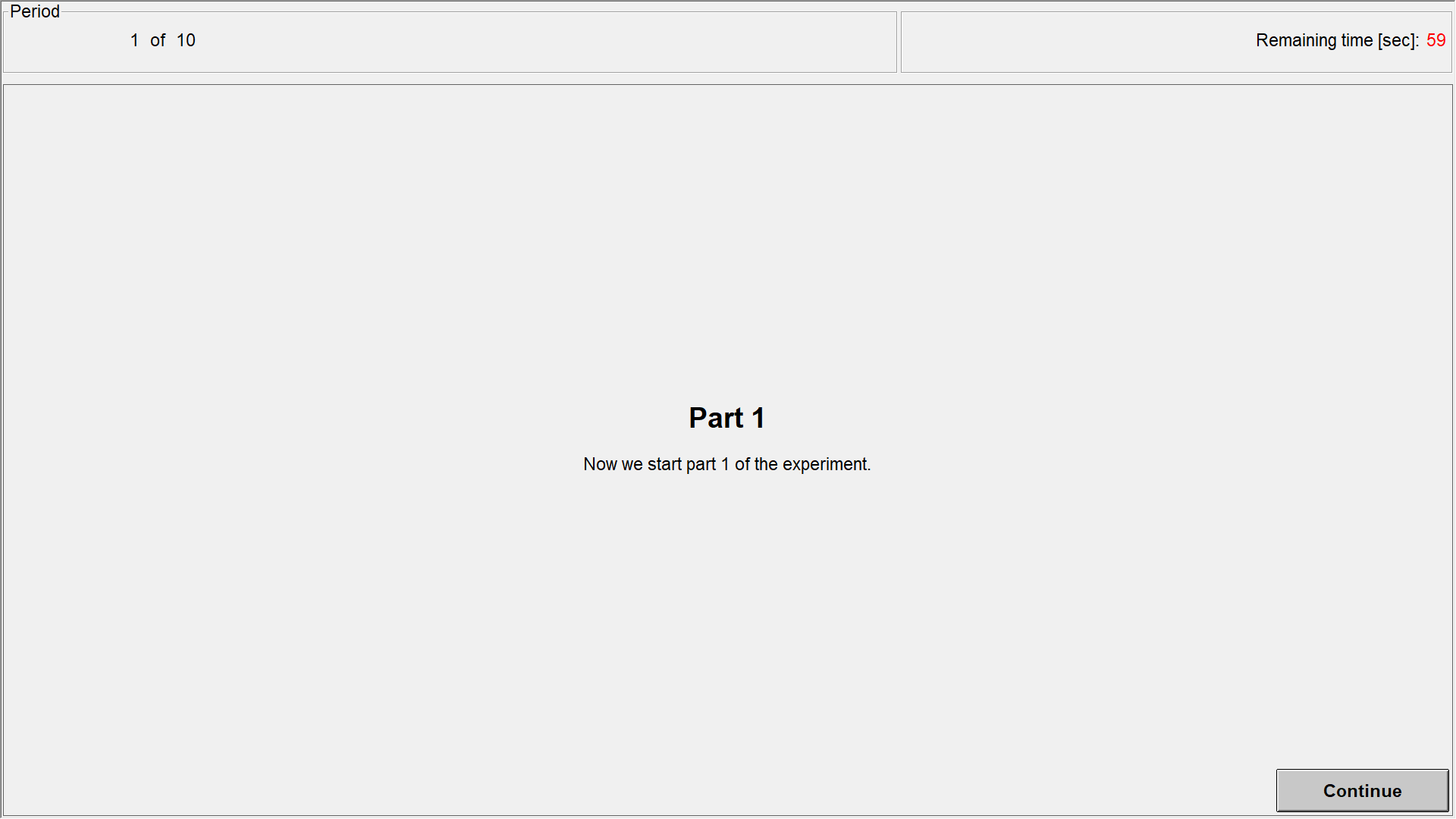


### Initial Social Anchors for Treatments L and LNSI (Period 1)


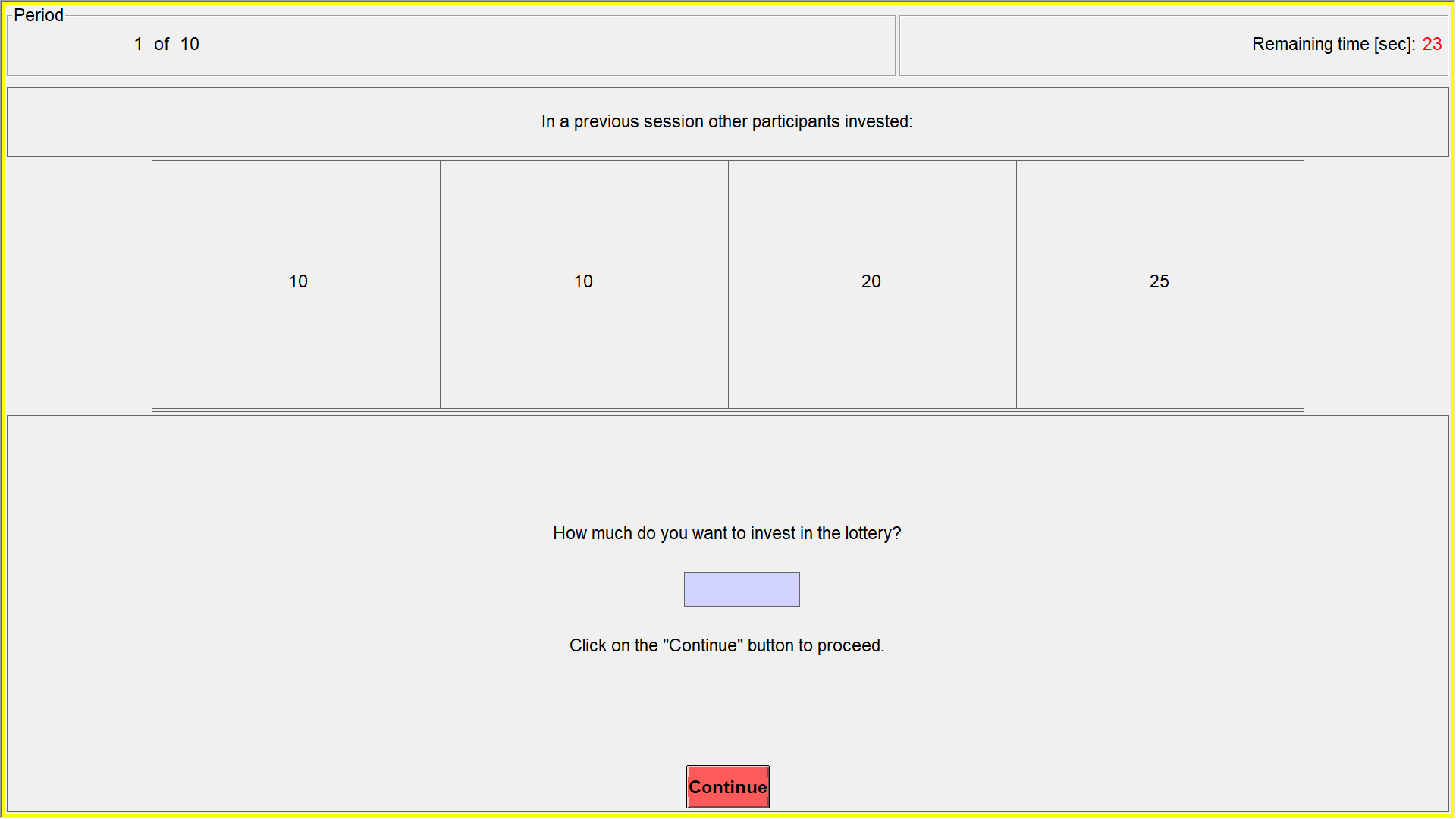


###

### Initial Social Anchors for Treatments H and HNSI (Period 1)


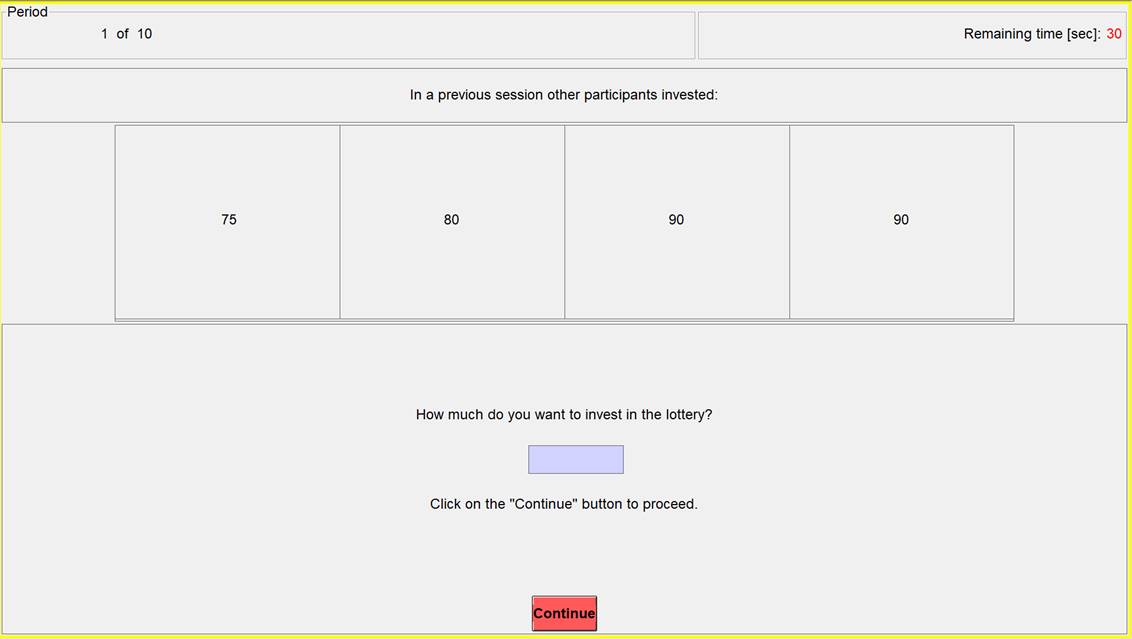


### Social Group Information in Treatments L and H: Periods $t\in\{2,\ldots, 10\}$.


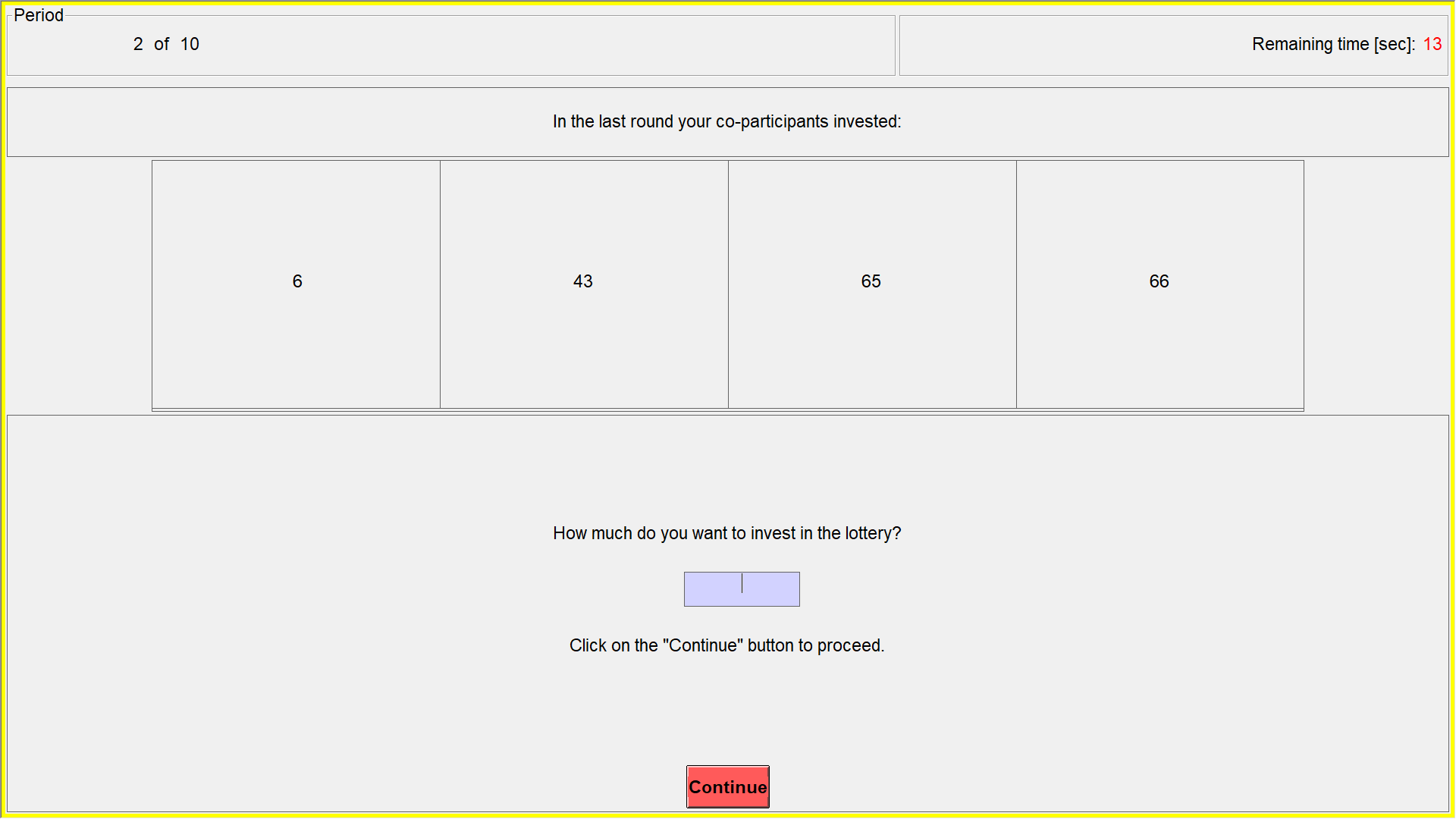


###

### No Social Group Information in Treatments LNSI and HNSI: Periods $t\in\{2,\ldots, 10\}$.


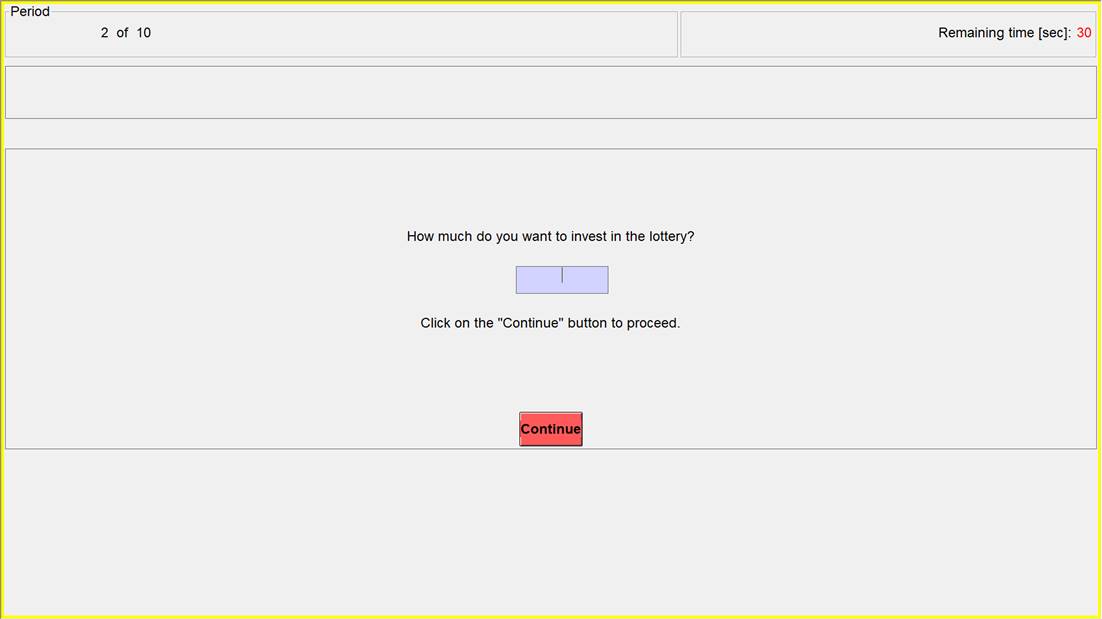


### Lottery Outcome After each Period


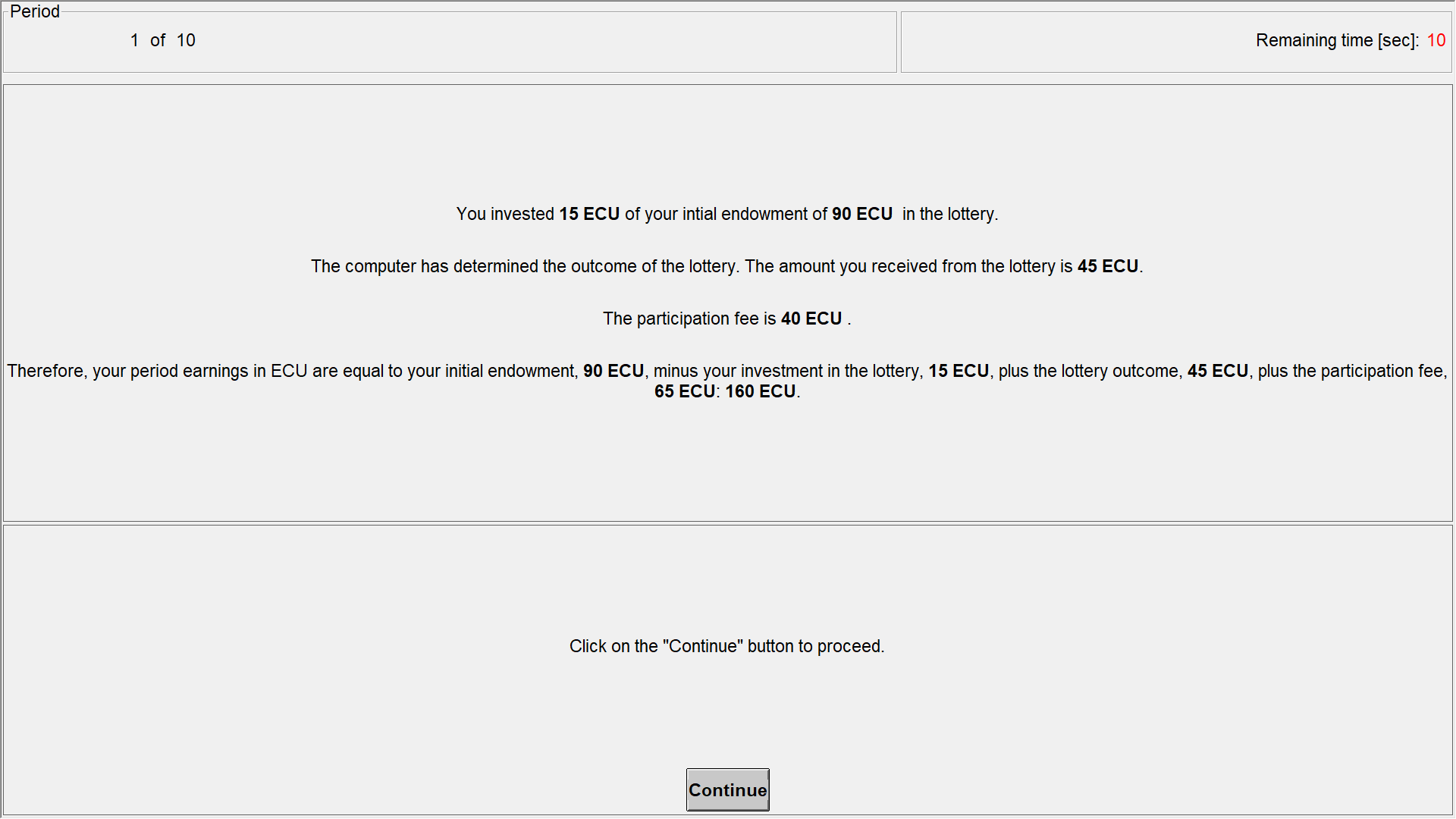


### Part 2


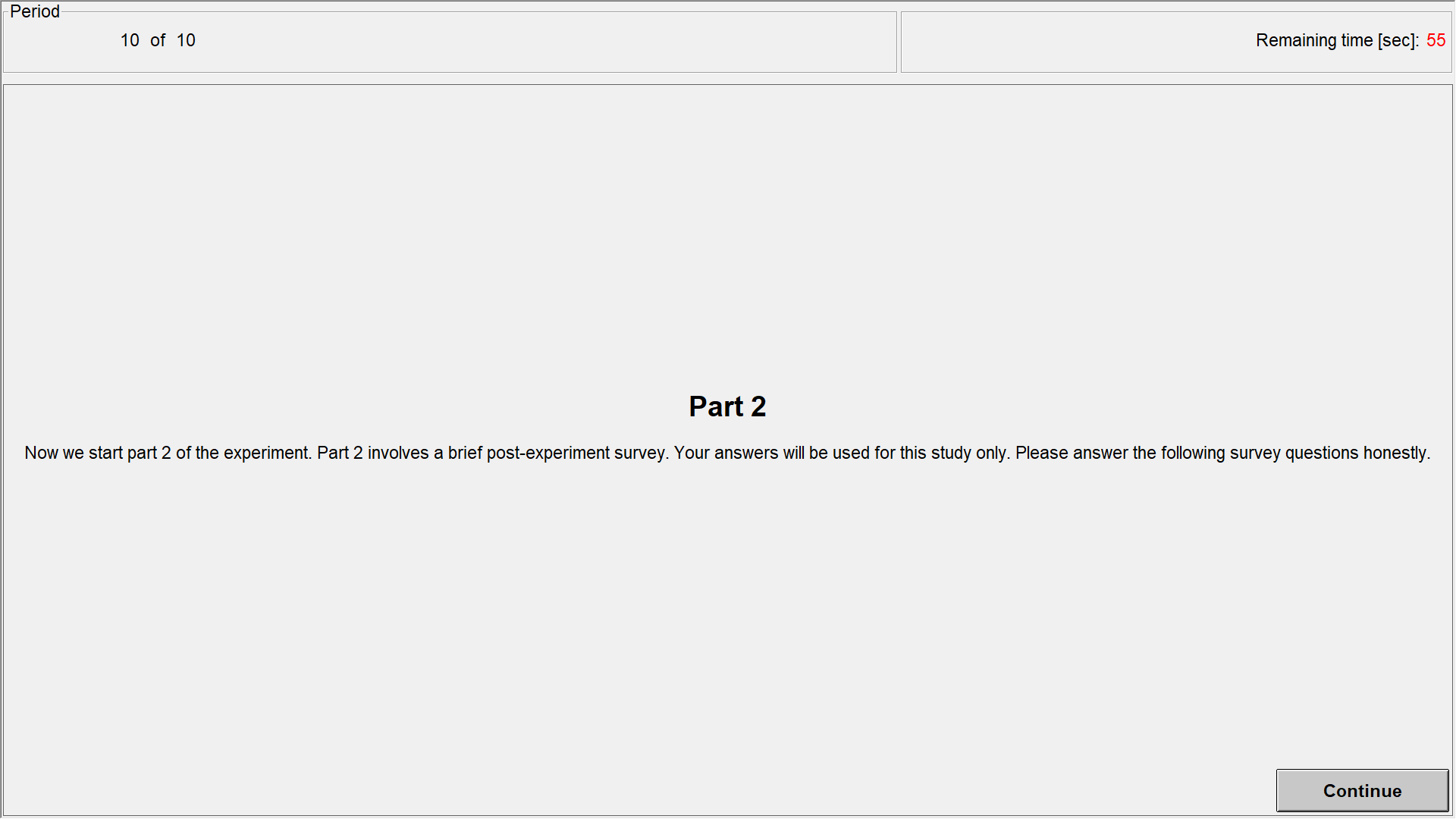


### Demographic Questions


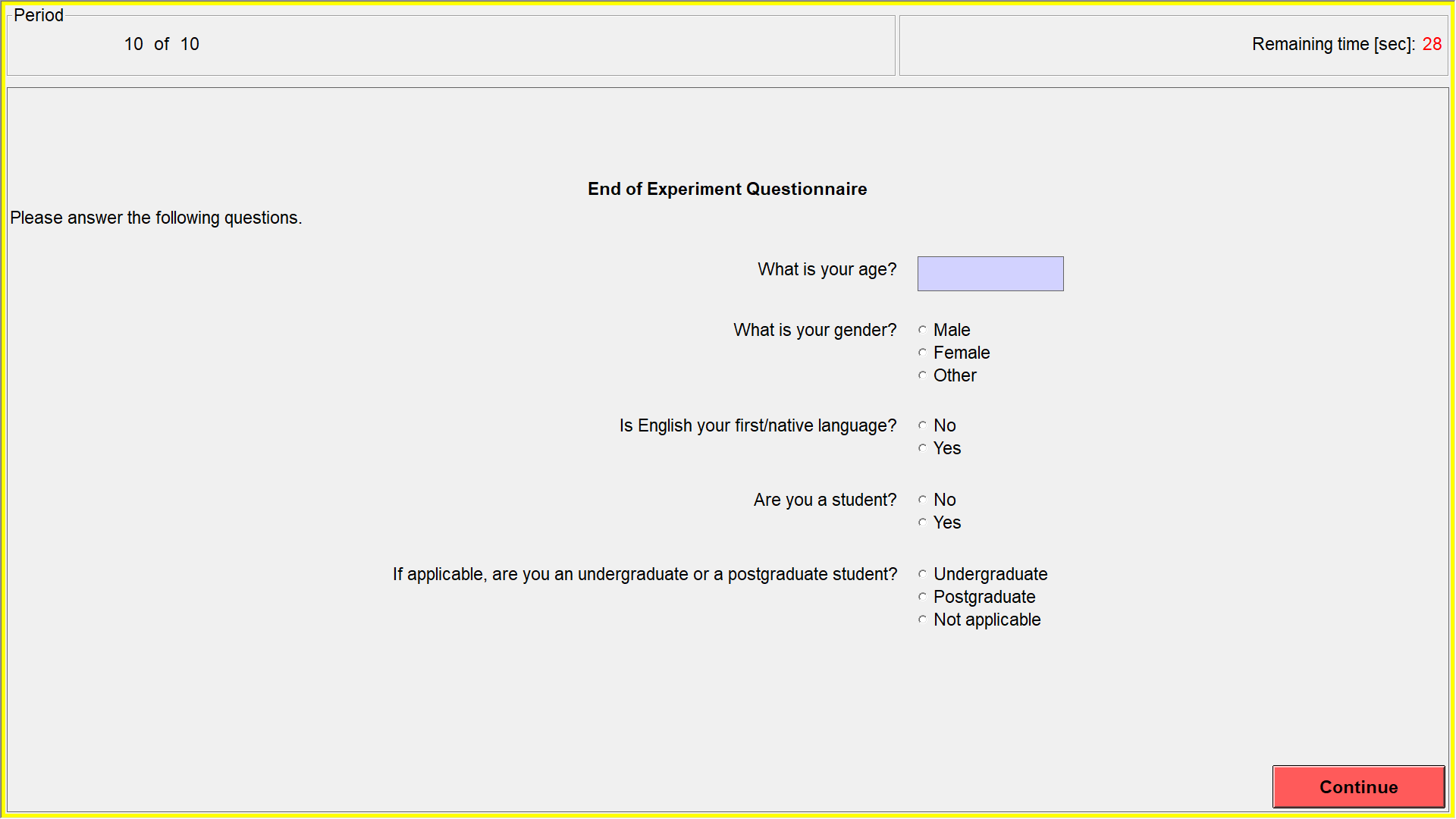


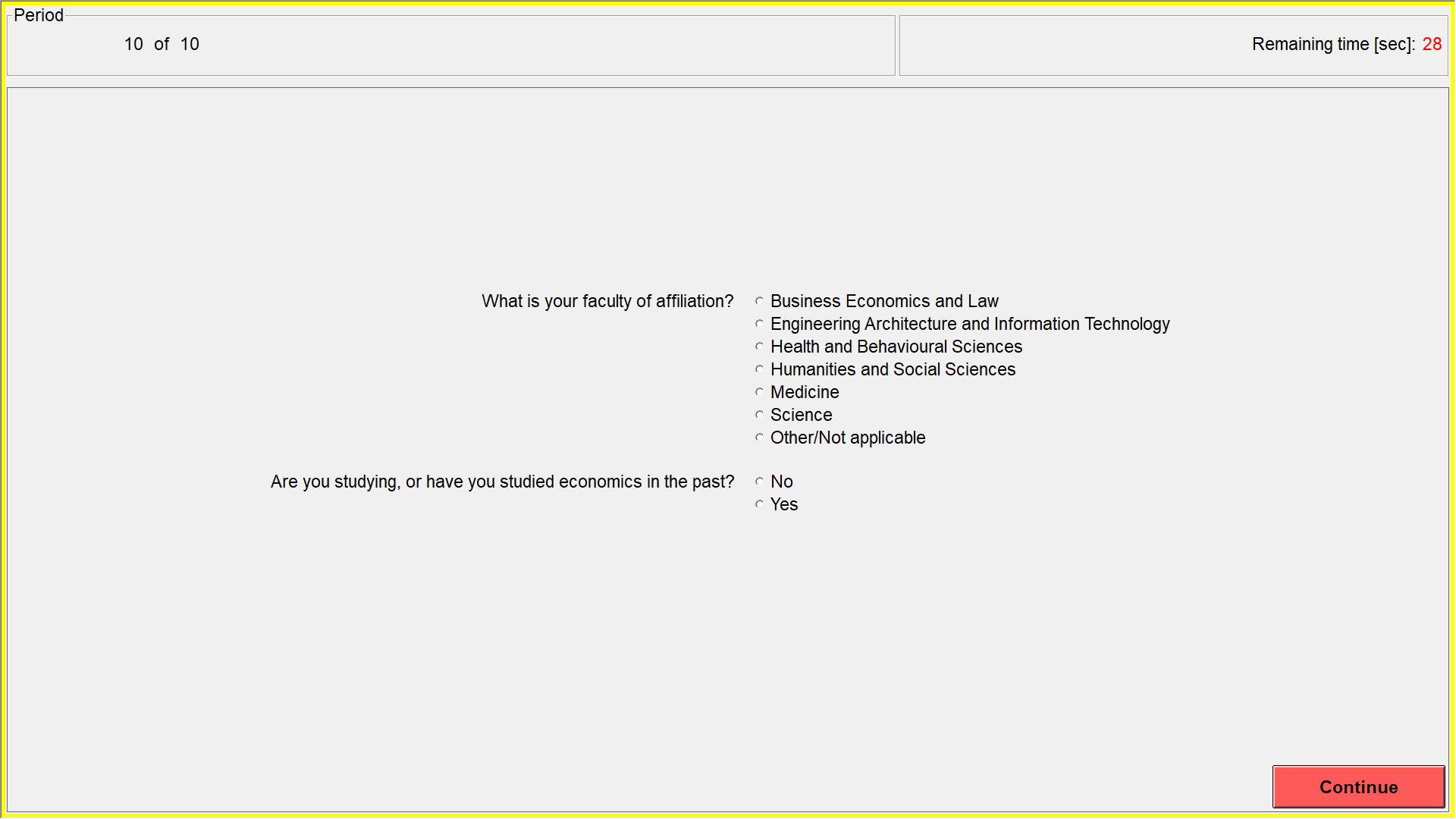


### Final Results


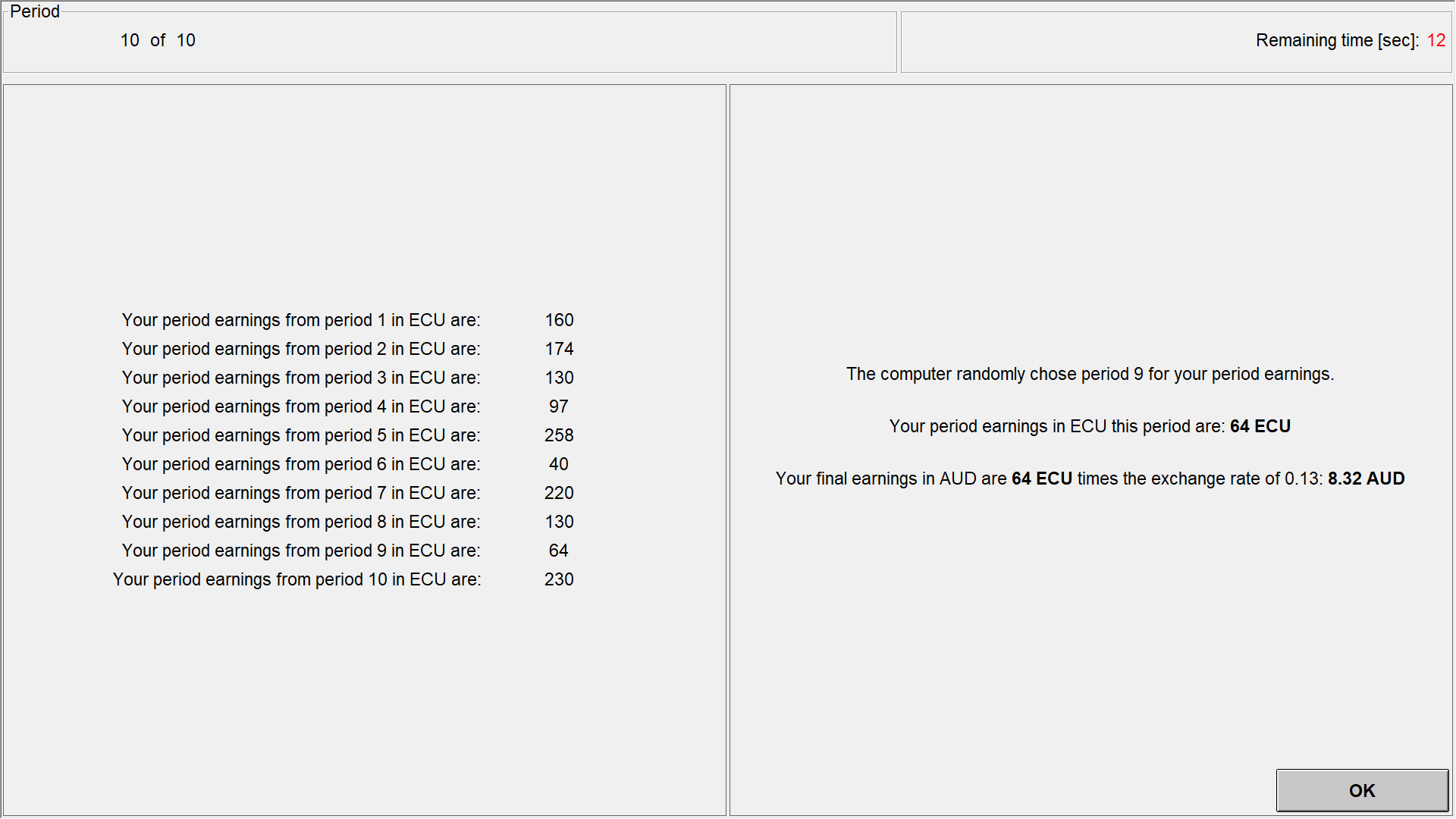


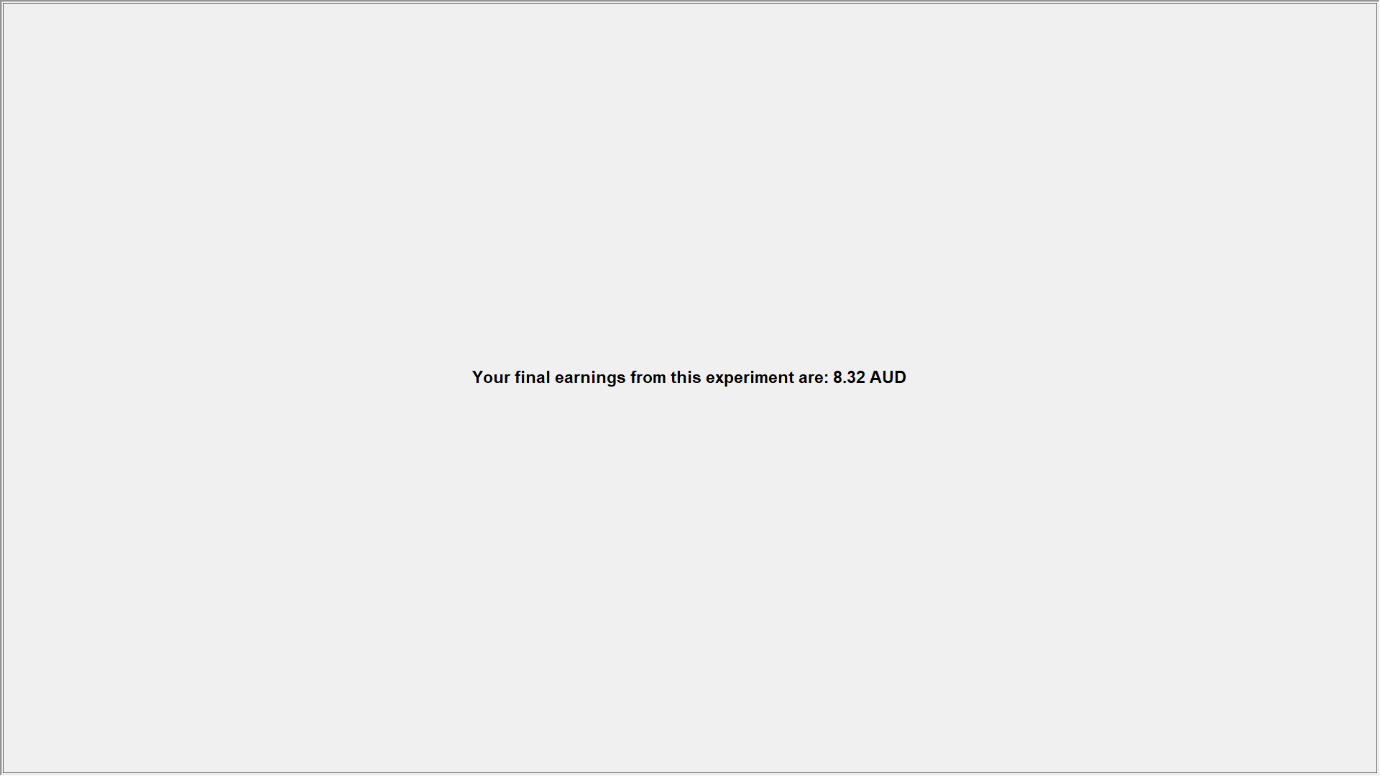


# A2. Additional Data Analysis

## A2.1 Preliminary treatment summary statistics

Table A2.1 provides a summary of the investment’s participants made in the one-round preliminary. With 31 observations collected, we find the mean investment to be slightly above the mid-point investment of 45 ECU. The minimum investment made in the treatment was 10 ECU and the maximum was 90 ECU. Table A2.2 provides a test of normality for the distribution of investments. We find no evidence of skewness in the data thus implying a normal distribution. Figure A2.1 provides a visual representation of the distribution of investment decisions. As mentioned in the methodology section of this thesis, a sample of investments were taken from this distribution and used as social anchors in the 2 x 2 factorial treatment design. For L and LNSI, we used investments {10, 10, 20, 25} (ECU), and for H and HNSI we used {75, 80, 90, 90} (ECU).

**Table A2.1:** Preliminary Treatment Summary Statistics (Investment)

| **Summary Statistics (Investment)** | | | | | | |
| --- | --- | --- | --- | --- | --- | --- |
| **Variable** | Observations | Mean | Std. Dev | Min | Max | |
| Investment | 31 | 46.3871 | 25.8001 | 10 | | 90 |

**Table A2.2:** Preliminary Treatment Test for Normality (Investment)

| **Test for Normality (Investment)** | | | | | | |
| --- | --- | --- | --- | --- | --- | --- |
| **Variable** | Observations | Pr(Skewness) | Pr(Kurtosis) | Joint | | |
|  |  |  |  | Adj chi2 | | Prob>chi2 |
| Investment | 31 | 0.4606 | 0.1385 | 2.99 | 0.2242 | |

**Figure A2.1:** Preliminary Treatment Frequency of Investment


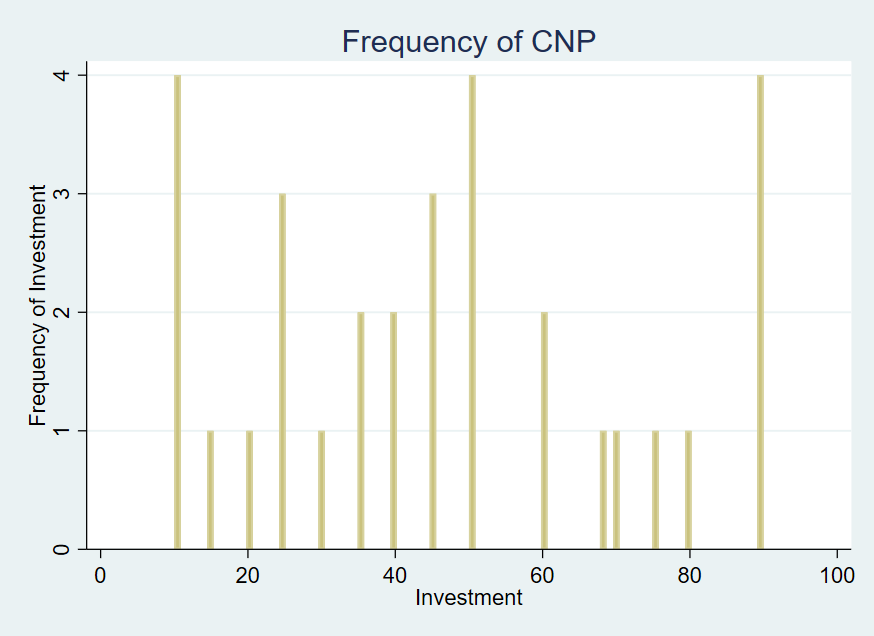


## A2.2 Demographic characteristics by treatment

Table A2.3 reports the demographic characteristics for our sample by treatment, including *χ^2^* on whether our sample’s key characteristics are equally distributed within our sample. In all cases, we find no statistical differences across our joint sample tests.

**Table A2.3:** Demographic characteristics by treatment

| **Treatment** | **N** | | **Female** | **Age** | **Undergraduate** | **Native speaker** | **Economics** |
| --- | --- | --- | --- | --- | --- | --- | --- |
| **L** | 70 | | .46 | 22.86 | .28 | .55 | .78 |
| **H** | 70 | | .57 | 23.68 | .5 | .54 | .73 |
| **LNSI** | 60 | | .56 | 22.47 | .33 | .56 | .70 |
| **HNSI** | 60 | | .56 | 22.78 | .38 | .42 | .73 |
| *Pearson χ^2^* | | | 0.46 | 0.73 | 0.15 | 0.31 | 0.73 |
|  | |  | | | | | |

## A2.3 Random effects Tobit regressions on investment rates over time

**Table A2.4:** Random effects Tobit regressions on investment rates over time

| Random Effects Tobit Models. Dependent variable: Investment  Periods: 1-10 | | | |
| --- | --- | --- | --- |
|  | **Model 1** | **Model 2** | **Model 3** |
| *L* | -12.441^**^  (5.179) | -28.882^****^  (5.740) | -30.443^****^  (5.554) |
| *LNSI* | -14.352^***^  (5.416) | -21.643^****^  (6.014) | -21.156^****^  (5.803) |
| *HNSI* | -0.717  (5.392) | 1.050  (5.981) | 1.543  (5.777) |
| *Period* | 1.379^***^  (0.174) | 0.308  (0.330) | 0.308  (0.330) |
| *L*Period* |  | 3.049^****^  (0.464) | 3.051^****^  (0.465) |
| *LNSI*Period* |  | 1.349^***^  (0.491) | 1.353^***^  (0.491) |
| *HNSI*Period* |  | -0.327  (0.484) | -0.326  (0.484) |
| *Female* |  |  | -14.50^****^  (3.749) |
| *English* |  |  | 3.602  (3.739) |
| *Economics* |  |  | 6.180  (4.715) |
| *SDS16 Score* |  |  | -1.144^**^  (0.556) |
| *Constant* | 54.31^****^  (3.780) | 60.02^****^  (4.064) | 72.01^****^  (8.421) |
| *N* | 2600 | 2600 | 2600 |
| *Wald χ^2^* | 74.19 | 138.74 | 165.52 |

**Notes:** Tobit regressions on investment censored at 0 and 90. Random effects control for the non-independence of investment choices within groups in L and H. Standard errors are presented in parentheses. ∗∗∗, ∗∗, ∗ indicate significance at the 0.1%, 1%, 5% and 10% level, respectively.

## A2.4 Frequency of investment by treatment

Figure A2.2 displays the frequency of investment for all periods by treatment. 700 investments were made in treatment L, 700 in treatment H, 600 in LNSI and 600 in HNSI.^[[1]](#footnote-1)^ A broad analysis of the four histograms indicate the initial social anchors could vary the mean and standard deviation of the investment distribution. If we firstly ignore the spike in investments at 90 ECU, there is a high probability the distribution of treatments L and LNSI are right skewed, as opposed to treatments H and HNSI which appear to be more uniform between 0 and 85 ECU. This suggests that the average investment for treatments that are exposed to low anchors are lower than those which receive high anchors.

**Figure A2.2:** Frequency of Investment by Treatment (All periods)

***
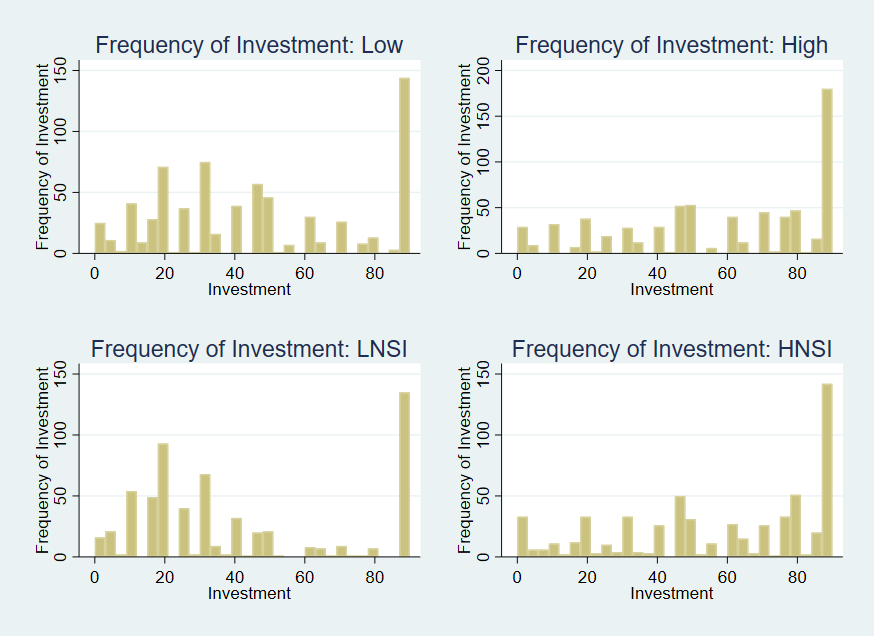
***

## A2.5 Multilevel mixed-effects linear regression (Treatments L & H)

In Table A2.5, we conduct a multilevel mixed-effects linear regression for treatments L and H. In the models, we set random effects at the group identity level (GroupID) and at the subject identity level (SubjectID). For this model we restrict the treatments to L and H, as treatments LNSI and HNSI have no social group information. Both models include two interaction terms between the dependent variables Treatment and Period and set treatment H as the base.

**Table A2.5:** Multilevel Mixed-Effects Linear Regression

| Multilevel mixed-effects linear regression: Dependent variable (Investment)  Periods: 1-10 | | |
| --- | --- | --- |
|  | Model 1 | Model 2 |
|  |  |  |
| *Treatment* |  |  |
| *L* | -26.93***  (4.797) | -21.72***  (5.159) |
|  |  |  |
| *Period* | -0.0410  (0.269) | 0.282  (0.314) |
|  |  |  |
| *Treatment*Period* |  |  |
| *L*Period* | 2.751***  (0.381) | 2.101***  (0.448) |
|  |  |  |
| *Constant* | 57.93***  (3.392) | 54.77***  (3.719) |
| *N* | 1400 | 1260 |
| *Wald Chi^2* | 108.71 | 61.87 |

Notes: Standard errors in parentheses. ^*^ *p* < 0.10, ^**^ *p* < 0.05, ^***^ *p* < 0.01

## A2.6 Frequencies of within-group standard deviations

**Figure A2.3:** Frequencies of within-group standard deviations


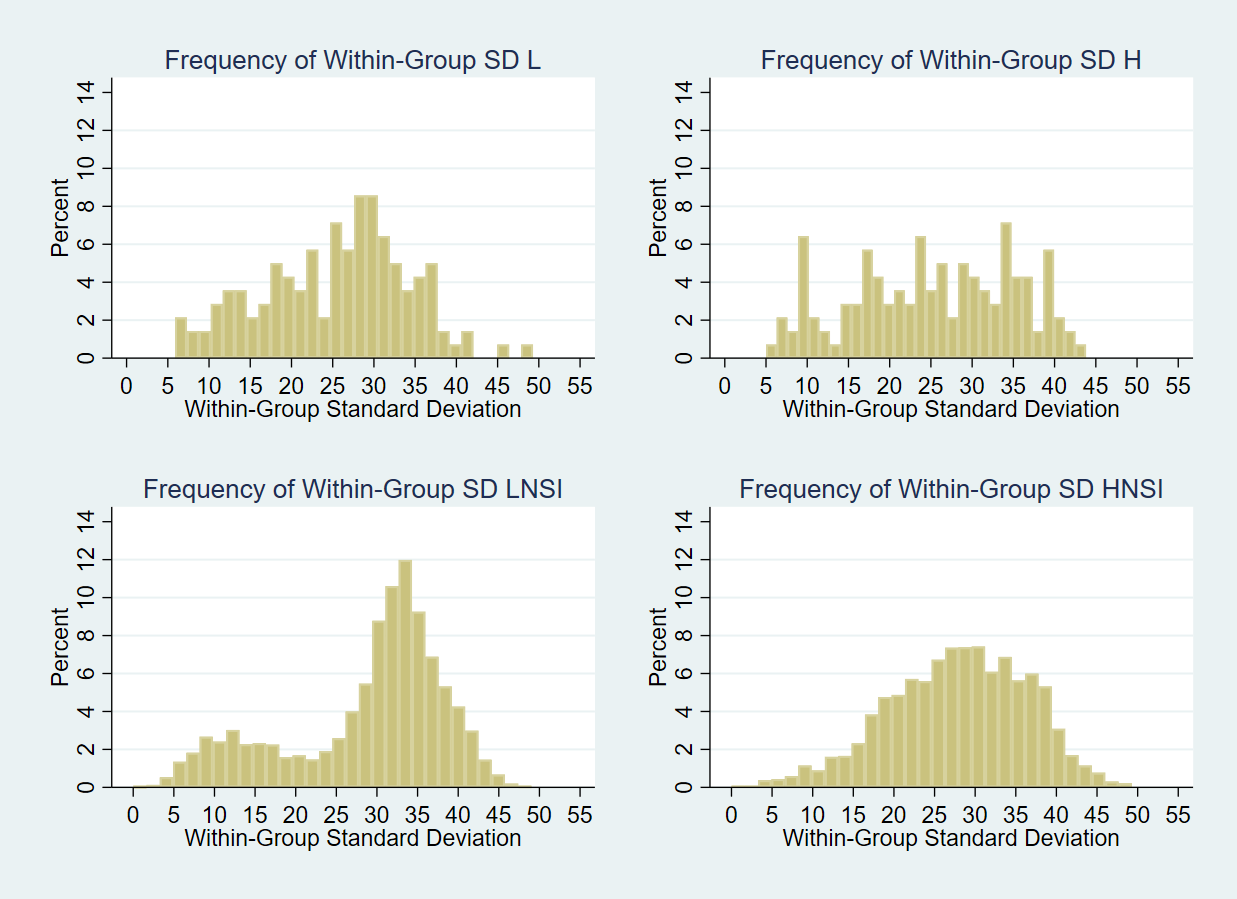


## A2.7 Investment change

In the spirit of the behavioral imitation models by Huck et al., (1999), Bigoni and Seutens (2012) and Liu et al. (2022), we analyze how subjects investment changes across rounds. Table A2.6 presents panel regressions on investment change in L and H as a function of either the social anchor (in round 1) or the social information (afterwards). We begin by estimating the following equation (Model 1), which verifies how the change in investment by agent *i* between time *t* ($x_{i}^{t}$) and time *t*-1 ($x_{i}^{t-1}$) is a function of the difference between $x_{i}^{t-1}$ and the mean investment $x_{-i}^{t-1}$ of agents other than *i* in the same group:

|  | $x_{i}^{t}-x_{i}^{t-1} =\beta_{0}+\beta_{1}(x_{i}^{t-1}-x_{-i}^{t-1})$ |  |
| --- | --- | --- |

**Table A2.6:** Panel regressions on investment change in L and H

| Fixed Effect Model. Dependent variable: Investment change | | | | | | | |
| --- | --- | --- | --- | --- | --- | --- | --- |
|  | **Model 1** | **Model 2** | **Model 3** | **Model 4** | **Model 5** | **Model 6** | **Model 7** |
| *Diff. in Investment:*  *own vs. others (t-1)* | 0.772***  (0.028) |  | 0.772****  (0.043) | 0.749****  (0.061) |  | 0.788****  (0.046) |  |
| *Invested less than others (t-1)* |  | 33.258****  (1.746) | 3.048  (2.386) | 3.062  (2.386) | 3.047 (2.389) | 1.084  (2.597) | 0.875  (2.601) |
| *Invested less than others (t-1) *Diff. Inv: own vs. others (t-1)* |  |  |  | -0.041 (0.091) |  |  |  |
| *Diff. in Investment:*  *own vs. others * L (t-1)* |  |  |  |  | 0.730****  (0. 0516) |  | 0.803****  (0.056) |
| *Diff. in Investment:*  *own vs. others * H (t-1)* |  |  |  |  | 0.731****  (0.041) |  | 0.777****  (0.055) |
| *Diff. in Investment:*  *own vs. others (t-2)* |  |  |  |  |  | 0.0186  (0.031) |  |
| *Diff. in Investment:*  *own vs. others * L (t-2)* |  |  |  |  |  |  | -0.0245  (0.043) |
| *Diff. in Investment:*  *own vs. others * H (t-2)* |  |  |  |  |  |  | 0.0671  (0.045) |
| *Constant* | 1.402**  (0.779) | -16.044***  (1.138) | -0.196  (1.390) | 0.298  (1.779) | -0.196  (1.391) | 0.983  (1.502) | 1.092  (1.504) |
| *N* | 1,260 | 1,260 | 1,260 | 1,260 | 1,260 | 1,120 | 1,120 |
| *F* | 731.65 | 362.79 | 366.85 | 244.46 | 244.35 | 231.26 | 139.25 |

**Notes:** Standard errors are presented in parentheses. ∗∗∗∗, ∗∗∗, ∗∗, ∗ indicate significance at the 0.1%, 1%, 5% and 10% level, respectively. Qualitatively similar results were found in models with robust standard errors.

In Model 1, the coefficient is positive and highly significant, in support of an imitation interpretation of our results. In Model 2, we introduce a dummy variable that takes the value of 1 if the participant in period t-1 invested less the other members of his group. The coefficient is positive and highly significant, in support of lagging behind one’s peers leading to an increase in investment in the following round. In Model 3, we test if both variables add explanatory power to investment change. Surprisingly, we find that the investment dummy becomes insignificant. In Model 4, we introduce an interaction between the two variables to explore if lagging behind one’s peer has a nonlinear effect investment change. However, the coefficient of the interaction dummy is also insignificant. In Model 5, we decompose the effect of investment difference by treatment and check whether there is an asymmetric effect across L and H. As both coefficients are hardly different from each other, we find no evidence for an asymmetric effect across treatments. In Models 6 and 7, we investigate whether further lagged investment of others has explanatory power on investment beyond what can be captured by the single lag model. We find it does not. Adjustment to information about investment of others is fully in place within the following period, leading to a simple Markov specification as captured by Model 1 being sufficient to capture the dynamics.

**References**

Bigoni, M., and Suetens, S. (2012). Feedback and Dynamics in Public Good Experiments. Journal of Economic Behavior and Organization, 82, 86–95.

Huck, S., Normann, H.-T., and Oechssler, J. (1999). Learning in Cournot oligopoly – an experiment. Economic Journal, 109, 80-95.

Liu, J., Sonntag, A. and Zizzo, D.J. (2022). Information Defaults in Repeated Public Good Provision, Journal of Economic Behavior and Organization, 197, 356-369.

##

## A2.8 Explaining investment decisions with OLS regressions

**Table A2.8:** OLS regressions on investment rates over time in L & H

|  | | | |
| --- | --- | --- | --- |
|  | **Model 1** | **Model 2** | **Model 3** |
| *Low* | -26.926^****^ (3.263) | -12.932^***^  (4.342) | -13.853^***^  (4.357) |
| *Low*Period* | 2.710^****^ (0.371) | 1.528^***^  (0.461) | 1.536^***^  (0.459) |
| *H*Period* | -0.041  (0.372) | 0.312  (0.437) | 0.311  (0.435) |
| *Others’ Investment Lag in t* - 1 |  | 0.316^****^ (0.051) | 0.313^****^  (0.052) |
| *Controls* | No | No | Yes |
| *Constant* | 57.93^***^ (2.307) | 37.278^***^  (4.165) | 27.88^***^  (11.29) |
| *N* | 1400 | 1260 | 1260 |
| *F* | 38.01 | 26.34 | 12.22 |

**Notes:** Our control variables are the following: gender, English as a first language, current or prior study of Economics and their average score on Stöber’s (2001) social desirability scale.

1. Total investment is a function of the number of participants in each treatment *n* and the number of periods *t*. [↑](#footnote-ref-1)
